# Violacein-Loaded Outer Membrane Vesicles from *Salmonella enterica* Exhibit Potent Anti-Melanoma Activity *in Vitro* and *in Vivo*

**DOI:** 10.1021/acsbiomaterials.5c00933

**Published:** 2025-09-11

**Authors:** Genesy Pérez Jorge, Marco Gontijo, Marina Flóro e Silva, Raquel Bester Liszbinski, Renata Spagolla Napoleão Tavares, Cyro von Zuben de Valega Negrão, Carlismari Oliveira Grundmann, Isabella Carolina Rodrigues dos Santos Goes, Lilian de Oliveira Coser, Elizabeth Bilsland, Francisca Janaína Soares Rocha, Monica Tallarico Pupo, Selma Giorgio, Sandra Martha Gomes Dias, Fausto Almeida, Marcelo Brocchi

**Affiliations:** a Departamento de Genética, Evolução, Microbiologia e Immunologia, Instituto de Biologia, 28132 Universidade Estadual de Campinas − UNICAMP, Campinas, São Paulo 13083-862, Brazil; b Research Group Statistics and Mathematical Modeling Applied to Educational Quality (GEMMA), University of Sucre, Sucre, Sincelejo 700001, Colombia; c Department of Molecular Genetics and Microbiology, Duke University, Durham, North Carolina 27710, United States; d Departamento de Biologia Animal, Instituto de Biologia, Universidade Estadual de Campinas - UNICAMP, Campinas, São Paulo 13083-862, Brazil; e Brazilian Biosciences National Laboratory (LNBio), Brazilian Center for Research in Energy and Materials (CNPEM), Campinas, São Paulo 13083-970, Brazil; f Graduate Program in Genetics and Molecular Biology, Institute of Biology, University of Campinas - UNICAMP, Campinas, São Paulo 13083-862, Brazil; g School of Pharmaceutical Sciences of Ribeirão Preto, 28133 University of São Paulo, Ribeirão Preto, São Paulo 14040-903, Brazil; h Departamento de Patologia Clínica, Faculdade de Ciências Médicas, Universidade Estadual de Campinas -UNICAMP, Campinas, São Paulo 13083-887, Brazil; i Synthetic Biology Laboratory, Department of Structural and Functional Biology, Institute of Biology, Universidade Estadual de Campinas -UNICAMP, Campinas, São Paulo 13083-862, Brazil; j Tropical Medicine Department, Medical Science Center, Federal University of Pernambuco, Recife, Pernambuco 50670-901, Brazil; k Department of Biochemistry and Immunology, Ribeirão Preto Medical School, University of São Paulo, São Paulo 14049-900, Brazil

**Keywords:** extracellular vesicles, *Salmonella enterica* Typhimurium, antitumor, macrophages, violacein, nanocarrier, delivery vector

## Abstract

Violacein exhibits antitumor activity, indicating potential for future clinical application. However, an efficient delivery system is required for the clinical use of this hydrophobic compound. Effective delivery systems can enhance the solubility and bioavailability of hydrophobic compounds like violacein, facilitating its clinical application for antitumor therapy. Recent studies have demonstrated that outer membrane vesicles (OMVs) can serve as nanocarriers. This article constitutes the first report to present both *in vivo* and *in vitro* investigations of OMVs derived from a hypervesiculating mutant of *Salmonella enterica* Typhimurium as a delivery vehicle for violacein. In this study, *S. enterica* Typhimurium Δ*tolRA* (with a hypervesiculated phenotype) was transformed with a plasmid encoding the violacein biosynthesis operon. OMVs and violacein-loaded OMVs were isolated, characterized, and used in the treatment of murine melanoma. We assessed the cytotoxic effect of these violacein-loaded OMVs in both two-dimensional (2D) and three-dimensional (3D) cell cultures. Violacein-loaded OMVs reduced melanoma cell viability (IC_50_: 9.30 × 10^8^ vesicles/mL) and delivered violacein in melanoma cells. Additionally, tumor regression was associated with treating tumor-bearing mice with violacein-loaded OMVs or nonviolacein-loaded OMVs (5 × 10^9^ vesicles/mouse). The antitumor response was linked to the accumulation of M1-type macrophages in the tumor microenvironment and the overexpression of mRNA for antitumor mediators Inducible Nitric Oxide Synthase, Tumor Necrosis Factor-alpha, and Interleukin-6 (iNOS, TNF-α, and IL-6). Our results suggest that OMVs can act as nanocarriers for highly hydrophobic agents and induce antitumor responses to eliminate tumors.

## Graphical abstract

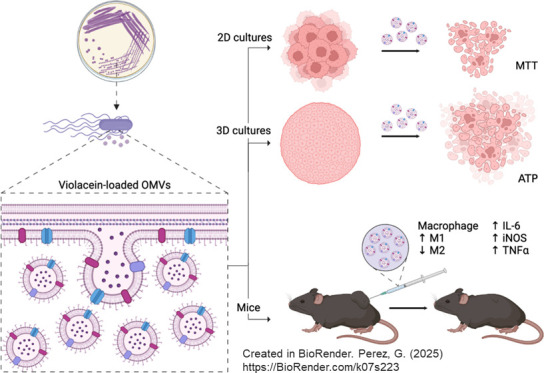

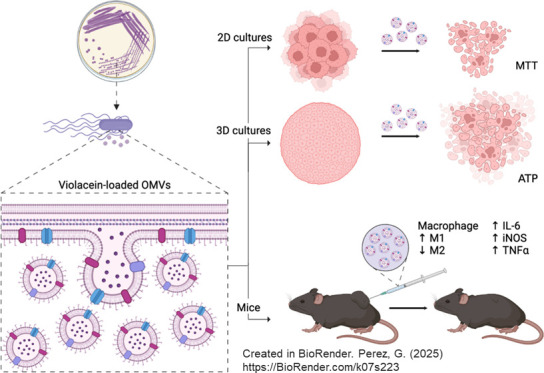

## Introduction

Melanoma is the most aggressive and metastatic form of skin cancer, and it leads the number of skin cancer deaths. According to the 2022 global cancer statistics, there were 331,647 new cases of melanoma and over 58,600 deaths worldwide, primarily due to its highly metastatic nature.^1^ Current treatment options for melanoma include surgical resection for early stages, often combined with chemotherapy, radiotherapy, or immunotherapy for metastatic stages.^2,3^ While these therapies have significantly improved survival and reduced mortality, many patients exhibit intrinsic resistance or experience tumor recurrence after initial treatment, with tumors often developing acquired resistance due to the disease’s heterogeneity.^4–6^ Consequently, there is an urgent need for novel therapeutic strategies to address melanoma effectively.

Violacein is a purple, hydrophobic secondary metabolite produced by various phylogenetically distinct Gram-negative bacteria.^7–12^ This pigment is an alkaloid belonging to the indocarbazole family and is synthesized through the oxidative dimerization of two tryptophan molecules.^13^ The genes responsible for violacein production are clustered in an operon known as *vioABCDE*.^14^ Violacein has been successfully overexpressed in bacteria of therapeutic interest, such as *Escherichia coli* and *Salmonella enterica* Typhimurium, by cloning the *vioABCDE* operon.^15^ This compound exhibits a broad spectrum of biological and pharmacological activities, including antibacterial, antifungal, antiviral, antiparasitic, and anticancer activity.^12,15–18^

Violacein’s anticancer activity is among its most promising pharmacological properties.^19^ Its cytotoxic effects have been demonstrated at nanomolar concentrations against various cancers, including melanoma, leukemia, colon, breast, and head and neck.^15,16,20–24^ Efficacy has been observed in murine models of Ehrlich ascites and head and neck tumors.^15,25,26^ Studies suggest that violacein induces cell cycle arrest and apoptosis by downregulating mdm2, AKT, and ERK, while upregulating Bax, p53, and p21.^22,24^ Violacein has recently been identified as an immunostimulant, activating the TLR pathway and inducing TNF-α (tumor necrosis factor-α) expression.^27^ Despite these promising results, the therapeutic use of violacein is constrained by its high hydrophobicity, which hampers its delivery to cancer sites.^28^

Bacteria and their derivatives offer promising sources of potential antitumor drugs.^29–31^ Evidence has shown that outer membrane vesicles (OMV) can potentially treat various types of cancer.^32–34^ OMVs comprise phospholipids, proteins, lipopolysaccharides, nucleic acids, and other small molecules, enabling them to interact with their environment and other bacterial and eukaryotic cells.^35–37^ Beyond their role as a delivery system, OMVs can also stimulate the immune system, as they contain components from the outer membrane of their parent bacteria.^30,33,35^

OMVs from Gram-negative bacteria such as *E. coli*, *Salmonella* Typhimurium, *Klebsiella pneumonia*, and *Fusobacterium nucleatum* have been explored as platforms for cancer therapy, particularly in colorectal and melanoma models.^38–41^ These vesicles have been engineered or surface-modified to enhance tumor targeting, reduce toxicity, and deliver chemotherapeutics or immunomodulators.^42^ For instance, OMVs expressing tumor ligands or checkpoint inhibitors have shown superior tumor accumulation and immune activation.^43^

In recent years, OMVs have gained increasing attention in oncology not only as immunostimulatory agents but also as versatile drug delivery platforms. Their intrinsic composition (rich in microbe-associated molecular patterns) allows OMVs to engage the immune system while delivering therapeutic cargos, making them ideal for use in cancer immunotherapy and nanovaccine design.^42^ Engineered OMVs have been successfully employed to display tumor neoantigens, promote dendritic cell maturation, and trigger robust cytotoxic T lymphocyte responses, significantly delaying tumor progression in preclinical models.^39^ Moreover, their membrane stability and capacity for surface modification enable the combination of OMVs with chemotherapeutic agents, immune checkpoint inhibitors, or gene-editing tools to enhance therapeutic outcomes.^39,42,44^ These advances underscore the versatility of OMVs across different bacterial strains and therapeutic contexts, supporting their development as scalable, multifunctional platforms in oncology.

Notably, OMVs loaded with apoptosis-inducing ligands related to human TNF can delay the progression, recurrence, and metastasis of cutaneous melanoma in murine models.^44^ Similarly, OMVs have been shown to efficiently deliver doxorubicin to tumor cells and inhibit tumor growth in A549 tumor-bearing mice.^39^ During the final writing of this study, a recently published manuscript reported that violacein administered through *Janthinobacterium lividum* vesicles retains its antitumor activity *in vitro*, underscoring the versatility of OMVs in preserving the efficacy of bioactive compounds.^45^

Despite this advance, key challenges persist in translating OMV-based violacein delivery to clinical settings. The inherent hydrophobicity of violacein demands robust encapsulation systems to ensure stability and targeted delivery. At the same time, the low natural yield of OMVs in wild-type strains like *J. lividum* limits scalable production.^45^ Recent studies have addressed these bottlenecks through genetic engineering strategies. For instance, mutations in the Tol-Pal system (deletions in *tolR* and *tolA*) induce a hypervesiculating phenotype in Gram-negative bacteria without compromising cell viability.^46,47^ The use of hypervesiculated mutants for the production of violacein-loaded OMVs offers a dual advantage: high-titer OMV production and cargo-loading capacity of hydrophobic drugs, thereby overcoming the scalability and drug delivery limitations observed in earlier systems.

Among Gram-negative bacteria, *S. enterica* has been extensively studied for cancer therapy due to its natural tumor targeting ability and strong immunostimulatory properties. These OMVs can activate the immune system through Toll-like receptors, particularly TLR4 and TLR5, reinforcing their potential as effective platforms for OMV-based cancer therapies.^29^ Genetic modifications in the Tol-Pal system, such as deletions in *tolR* and *tolA* *genes*, have been successfully applied in *Salmonella* to enhance OMV production without compromising bacterial viability or immunogenicity, supporting its suitability as a framework for OMV-based cancer therapies.^48^

In this study, we utilized an attenuated mutant of *S.* Typhimurium Δ*tolRA* to prepare violacein-loaded OMVs. We assessed the cytotoxic effect of these violacein-loaded OMVs in both 2D and 3D cell cultures, comparing them with OMVs without violacein and purified violacein. The safety of OMVs treatment was also evaluated in mice. Additionally, we examined the anticancer effects of OMVs and the associated immune response in a murine melanoma model. Our findings suggest that violacein-loaded OMVs effectively deliver violacein into tumor cells and elicit a robust antitumor immunity, leading to melanoma regression.

## Materials and Methods

### Bacterial Strains, Plasmids, and Growth Conditions

In this study, we utilized *S.* Typhimurium ATCC 14028 and the double hypervesiculated mutant Δ*tolRA* constructed and characterized previously.^49,50^ Both bacterial strains were transformed by electroporation with the plasmid pBAT_*vioABCDE*, resulting in the strains 14028 pBAT_*vioABCDE* and 14028 Δ*tolRA* pBAT_*vioABCDE*, respectively.^17^ The bacteria were cultured aerobically at 37 °C in Luria–Bertani (LB) broth and LB agar medium. Ampicillin was used at a concentration of 100 μg/mL, or kanamycin at 50 μg/mL, as required.

### Violacein

The violacein used in this study was obtained from Sigma-Aldrich (St. Louis, MO, USA).

### *In Vitro* Growth of Bacterial Strains

The *in vitro* growth of recombinant bacterial strains was performed in LB broth as previously described.^51^ Strains were plated on LB agar and incubated at 37 °C for 16 h. A single colony was then inoculated into LB broth and incubated for 16 h at 37 °C with shaking at 150 rpm. The following day, the cultures were diluted 1:100 in LB medium prewarmed to 37 °C and incubated under the same conditions. Bacterial growth was monitored over 12 h by collecting aliquots to measure optical density (OD) at λ600 and determining colony-forming units (CFU) through serial dilutions and the microdroplet technique on LB agar plates.^52^ Three independent experiments were performed.

### Isolation of OMVs

OMVs from *S. enterica* Typhimurium 14028, *S. enterica* Typhimurium 14028 Δ*tolRA*, *S. enterica* Typhimurium 14028 pBAT_*vioABCDE,* and *S. enterica* Typhimurium 14028 Δ*tolRA* pBAT_*vioABCDE* were isolated following a previously described protocol.^53^ Bacteria grown for 16 h at 37 °C were diluted 1:100 in fresh LB broth and incubated until reaching exponential growth phase (OD_600_ = 0.6). Violacein expression was then induced with IPTG to a final concentration of 1 mM, and the cultures were incubated for an additional 16 h at 37 °C. One milliliter of these cultures was reserved for CFU counting. The remaining cultures were centrifuged at 4 °C and 5000 × g for 45 min. The supernatants were filtered through a 0.22 μm filter and concentrated using 100 kDa ultrafiltration membranes (Millipore Amicon, Billerica, MA, USA). The supernatants were centrifuged at 13000 × g for 15 min at 4 °C and filtered again through a 0.22 μm filter. The supernatants were ultracentrifuged at 4 °C and 60,000 rpm (Beckman Coulter Optima L-90K ultracentrifuge) for 90 min. Purified OMVs samples were resuspended in 1× PBS, filtered again through a 0.22 μm filter, aliquoted, and stored at −80 °C until further use. The OMV concentration, based on total protein content, was determined using the Bradford method. The isolated OMVs were designated as follows: OMVs from *S. enterica* Typhimurium 14028 as ST-OMV; OMVs from *S. enterica* Typhimurium 14028 Δ*tolRA* as *tolRA*-OMV; OMVs isolated *S. enterica* Typhimurium 14028 pBAT_*vioABCDE* as ST*vio*-OMV; and OMVs isolated from *S. enterica* Typhimurium 14028 Δ*tolRA* pBAT_*vioABCDE* as *tolRAvio*-OMV.

### Characterization of OMVs

#### Nanoparticle Tracking Analysis (NTA)

The size, distribution, and particle count of the OMV preparations were analyzed using nanoparticle tracking analysis (NTA) with the NanoSight NS300 system (Malvern Instruments, Malvern, UK). OMV samples were diluted in 1× PBS and introduced into the NanoSight for rapid video capture. The focus, camera settings, and brightness were adjusted to ensure the vesicles were visible. Videos, each 60 s in duration, were recorded in triplicate and analyzed using NanoSight software (version 3.2.16).

#### Transmission Electron Microscopy (TEM)

For TEM imaging, 20 μL of OMV samples were applied to a copper grid and stained with 2% (w/v) uranyl acetate in maleate buffer for 4 min. After the grid dried, the OMVs were examined using a Zeiss Transmission Electron Microscope (LEO-906) at an accelerating voltage of 60 kV.

#### Detection and Quantification of Violacein in OMVs Using HPLC

OMVs were isolated using the previously described methodology. Violacein was purified as outlined in earlier studies.^37,54^ The violacein was extracted twice from the OMVs with ethyl acetate, with extraction occurring overnight. The crude violacein extracted with ethyl acetate was further analyzed by analytical HPLC using a Shimadzu Prominence system. This system was equipped with an SPD-M20A DAD diode array detector, an ELSD-LT II evaporative light scattering detector, an LC- 20 AD pump, a CBM-20A system controller, a CTO-20A column oven, a SIL-20A injector, and a SIL-20A autosampler. The analysis employed a C18 reversed-phase column (Ascentis, C18, 2.7 μm, 100× 4.6 mm) and LabSolution software. The gradient consisted of acetonitrile and water with a 1 mL/min flow rate. Violacein (Sigma-Aldrich, USA) was used as a standard. Three independent experiments were performed.

### Cancer Cell Lineage

The B16F10 murine melanoma cell line was cultured in Dulbecco’s Modified Eagle Medium (DMEM, Sigma-Aldrich, USA) supplemented with 10% fetal bovine serum (Vitrocell, BR) (v/v), penicillin (100 U/mL), and streptomycin (100 μg/mL) (Sigma-Aldrich, USA), Cultures were maintained at 37 °C in a humidified atmosphere with 5% CO_2_. Cells were subcultured every two to 3 days using 0.1% trypsin (Sigma-Aldrich, USA).

### Cell Viability Test and Determination of the 50% Inhibitory Concentration of OMVs in 2D Culture

Cytotoxicity was assessed using the MTT assay ([3-(4,5-dimethylthiazol-2-yl)-2,5-diphenyltetrazolium bromide], Sigma-Aldrich, USA), which measures the reduction of tetrazolium salts to formazan crystals.^55^ Cells were seeded into a 96-well plate at a density of 1 × 10^4^ cells/well and allowed to adhere overnight. The OMVs (ST-OMV, *tolRA*-OMV, ST*vio*-OMV, *tolRAvio*-OMV) were diluted in a medium without serum and antibiotics. Cells were then treated with a range of different dilutions of OMVs (starting with 2.036 × 10^10^ vesicles/mL) for 24 h. SDS (sodium dodecyl sulfate) at 150 μg/mL was included as a killing control. Each dilution was performed two times in sextuplicate.

The medium was then replaced with MTT solution (1 mg/mL) and incubated for 4 h. Following incubation, the medium was removed, and 200 μL of acidified isopropanol (0.04 M HCl) was added to each well. The plate was then homogenized for 20 min, and absorbance was measured at 570 nm using an Enspire microplate reader (PerkinElmer, USA). The percentage of viable cells was calculated using the following formula:

(%)=100(Sampleabsorbance)/(Controlabsorbance)

Cell viability data were obtained from three independent experiments (*n* = 3) and normalized to the untreated control.

### Optical Microscopy of B16F10 Cells Treated with OMV

The effect of OMVs on B16F10 cells was examined using optical microscopy. Cells (1 × 10^5^) were plated in 24-well plates with 13 mm diameter round glass coverslips and incubated for 16 h under standard culture conditions. OMVs at a 5 × 10^9^ vesicles/mL concentration were added to each well and incubated for 24 h at 37 °C with 5% CO_2_. Three wells were used for each type of OMV, and untreated cells served as control. Following treatment, B16F10 cells were fixed with methanol (Synth 99.8%) for 30 min, washed with 1× PBS, and stained with Giemsa stain (analytical Fluka) in a buffer of 0.07 M KH_2_PO_4_ and 0.07 M Na_2_HPO_4_ buffer. The coverslips were then mounted on glass slides and examined microscopically. The experiment was performed in triplicate and repeated in two independent experiments.

### Three-Dimensional (3D) Model of Toxicity

The cytotoxicity of the vesicles was also assessed using a three-dimensional (3D) B16F10 cell model. To prevent cell adhesion to the well surfaces and to promote cell aggregation, 2 × 10^4^ cells/well were seeded in U-shaped 96-well plates (Greiner Bio-One, Austria) previously coated with poly­(2-hydroxyethyl methacrylate) (poly-HEMA, Sigma-Aldrich, USA). After 72 h, the cell aggregates were exposed to three concentrations of OMV, with the highest concentration being 2.036 × 10^10^ vesicles/mL, for 24 h. The viability of the aggregates was then evaluated using the CellTiter-Glo 3D kit (Promega, USA), which measures ATP from viable cells through a reaction with luciferin and luciferase to produce a luminescent signal. Luminescence was measured using a CLARIOstar Plus microplate reader (BMG Labtech, Germany).

### Fluorescence Microscopy

Violacein is a purple fluorescent pigment that absorbs at 570 nm and emits at 675 nm. B16F10 cells were plated into a 96-well plate at a density of 1 × 10^4^ cells/well and allowed to adhere overnight. The following day, cells were treated with serial dilutions of OMVs and incubated for 4 h. Cell images were then analyzed using a High-Content Analysis Operetta 3.5 (PerkinElmer, USA) at 20× magnification. Additionally, in the 3D toxicity assay, the aggregated cell plates were examined after 24 h using the same microscope at 2 × and 10 × magnification.

### Quantification of Violacein by Optical Density

Based on the absorption spectra of violacein, we measured the optical density of vesicles containing violacein and compared it with the absorbance of a known concentration of violacein. Measurements were taken at 570 nm using an Enspire plate reader (PerkinElmer, USA). Additionally, using the concentration of violacein in the OMVs determined by HPLC, we calculated the violacein concentration corresponding to the Half-Maximal Inhibitory Concentration (IC_50_) value obtained from the cytotoxicity assay.

### Mice

C57BL/6JUnib mice (female, 6–8 weeks old) were obtained from the Multidisciplinary Center for Biological Research (CEMIB – UNICAMP). These mice were housed in ventilated mini-isolators (ALESCO, São Paulo, Brazil) under specific pathogen-free conditions with a standard laboratory environment of 24 ± 2 °C, a 12 h light/dark cycle, and 50 ± 10% relative humidity. They had *ad libitum* access to water and a solid diet (Nuvilab, Colombo, PR, Brazil). Mice were acclimated in our laboratory for 2 weeks prior to the start of the experiment. To facilitate subcutaneous inoculations and tumor growth monitoring, mice between 8 and 9 weeks old were shaved on the right flank 2 days before the initial inoculation. All experimental procedures involving animals were conducted in accordance with the guidelines and regulations of the Animal Research Ethics Committee of the State University of Campinas. The study protocols were approved under numbers 5769–1/2021, 5820–1/2021, and 5895–1/2021. The procedures complied with the ethical standards established in the ACS Ethical Guidelines to Publication of Chemical Research.

### Evaluation of the Safety Profile of OMV Treatments

Mice were randomly assigned to 7 groups, each consisting of 5 mice. Group I served as the PBS control and received subcutaneous (sc) injections of 60 μL of PBS 1× twice a week for 2 weeks. Group II received sc injections of 2 × 10^10^ *tolRA*-OMV vesicles twice weekly for 2 weeks. Group III was treated with 1 × 10^10^ *tolRA*-OMV vesicles, while Group IV received 5 × 10^9^ vesicles *tolRA*-OMV twice a week for 2 weeks. Group V was administered 2 × 10^10^ *tolRAvio*-OMV vesicles sc twice weekly for 2 weeks. Group VI received 1 × 10^10^ *tolRAvio*-OMV vesicles sc twice weekly for 2 weeks. Group VII was treated with 5 × 10^9^ *tolRAvio*-OMV vesicles twice weekly for 2 weeks. All mice were weighed three times a week throughout the experimental period and monitored for signs of illness, including eye discharge, diarrhea, pain, piloerection, or lethargy. One week after the final dose, the animals were euthanized by intraperitoneal injection of 5 mg/kg xylazine and 40 mg/kg ketamine, followed by cervical dislocation. Major organs (liver, spleen, kidney, and lungs) from mice in the 5 × 10^9^ vesicles groups were collected, fixed in 4% paraformaldehyde, and processed for histopathological analysis using hematoxylin and eosin (H&E) staining. Tissues were examined with a light microscope (Leica DM5500 B, Wetzlar, Hesse, Germany). Two independent experiments were performed.

### Assessment of the Antitumor Effect

C57BL/6JUnib mice were inoculated sc with 3 × 10^6^ B16F10 tumor cells in the right flank. When the average tumor size reached approximately 100 mm^3^ (10–12 days postinoculation), the tumor-bearing mice were randomly assigned to one of four groups (seven mice per group) for treatment: PBS control, violacein (at the same concentration as in the OMV), 5 × 10^9^ *tolRA*-OMV, and 5 × 10^9^ *tolRAvio*-OMV. Treatments were administered intratumorally twice a week for 2 weeks, at 60 μL per treatment. Tumor dimensions were measured using a caliper, and tumor volume (in mm^3^) was calculated using the formula:

$$\mathrm{Tumor}\;\mathrm{volume}=\left({\mathrm{Width}}^{2}\right)\times \left(\mathrm{Length}\right)\times 1/2$$

Tumor weight and volume were measured every 2–3 days throughout the experiment. After the treatment period, the tumor, liver, spleen, kidney, and lung were harvested and stained with hematoxylin and eosin for histopathological analysis. The experiment was conducted in duplicate.

### Antitumor Response Induced by OMVs or Free Violacein

C57BL/6JUnib mice were used to investigate the antitumor immune response elicited by *tolRA*-OMV and *tolRAvio*-OMV. When tumors reached approximately 100 mm^3^ (10–12 days after inoculation), mice were intratumorally administered 5 × 10^9^ vesicles, 306 ng of violacein (equivalent to the amount present in the OMV), or PBS. Four days following the first treatment, mice were given a second dose under the same conditions. Four hours after the second inoculation, the mice were euthanized. The tumor tissue was divided into two parts: one portion was used for flow cytometry to analyze macrophage populations, while RNA was extracted from the other for gene expression analysis related to the antitumor response.

### Flow Cytometry

The tumor mass was collected and washed with ice-cold PBS. To prepare a cell suspension, the tumor was cut into small pieces and digested with collagenase IV at 37 °C for 1 h. The resulting suspension was then filtered through a 70 μM cell strainer.

The isolated cell suspension was incubated in the dark at 4 °C for 20 min with the following antibodies against macrophage phenotype markers (CD11b-PerCP, F4/80-FITC, CD80-PE, and CD206-APC (Ebioscience, Houston, USA)). After incubation, the cells were washed with 1% BSA in PBS and centrifuged at 400 × g at 4 °C for 5 min. The supernatant was discarded, and the cells were resuspended in 2% paraformaldehyde solution and incubated for 20 min at 4 °C in the dark. Flow cytometry data were acquired on a NovoCyte flow cytometer (ACEA Bioscience, USA), collecting 50,000 events per sample. The panel included macrophages (F4/80+ CD11b+), M1 macrophages (F4/80+ CD80+), and M2 macrophages (F4/80+ CD260+). Data analysis was performed using NovoExpress 1.5.0 software.

### Quantitative RT-PCR (qRT-PCR)

The tumor mass was macerated in liquid nitrogen, and total RNA was extracted using the Direct-zol RNA MiniPrep Plus kit (Zymo Research. Irvine, CA, USA) and Trizol reagent (Invitrogen, USA), following the manufacturer’s instructions. RNA integrity was assessed using a 1% agarose gel stained with ethidium bromide. RNA concentration and purity were analyzed with a NanoDrop 2000c (Thermo Scientific, USA). 1.5 μg of RNA from each sample was treated with DNase I Amplification grade (Sigma-Aldrich. Louis, MO, USA) to remove any contaminating genomic DNA. PCR was performed on the DNase-treated samples to confirm the absence of genomic DNA contamination. Reverse transcription was carried out using the High-Capacity cDNA Reverse Transcription kit (Applied Biosystems. USA) following the manufacturer’s instructions. The cDNA was diluted 1:10. Quantitative PCR was performed using the 2x qPCRBio SyGreen Mix Separate-Rox kit (PCRBIOSYSTEMS, Wayne, Pennsylvania, USA) with the StepOnePlus Real-Time PCR System (Applied Biosystems, USA). Primers for Bax, VEGF, IL-6, iNOS, *K*_i_-67, and TNF-α were used as described previously.^50^ Primer specificity and quality were verified by analyzing the dissociation curves. Gene expression levels were normalized to β-actin and GAPDH, and relative expression was calculated using the 2^–ΔΔCT^ method.^56^

### Statistical Analysis

Data are presented as mean ± standard deviation. One-way ANOVA was used to compare more than two groups, while a two-tailed Student’s *t* test was used to compare two groups. All statistical analyses were conducted with the GraphPad Prism version 8.0.1 for Windows (GraphPad, San Diego, CA, USA).

## Results

### Deletion of the *tolR* and *tolA* Genes Increases the Production of OMVs

In this study, OMVs were prepared from a hypervesiculated and attenuated mutant of *S. enterica* Typhimurium 14028 Δ*tolRA*. *S. enterica* Typhimurium 14028 Δ*tolRA* and *S. enterica* Typhimurium 14028 strains were transformed with the plasmid pBAT_*vioABCDE*. After 24 h, purple colonies of varying intensities were observed in both strains (Figure S1A).

To investigate the effect of violacein production in *S. enterica* Typhimurium, we compared the *in vitro* growth of *S. enterica* Typhimurium 14028, *S. enterica* Typhimurium 14028 pBAT_*vioABCDE*, *S. enterica* Typhimurium 14028 Δ*tolRA,* and *S. enterica* Typhimurium 14028 Δ*tolRA* pBAT_*vioABCDE* strains. Growth was monitored for 12 h using OD_600_ measurements, and CFU counts on LB agar plates (see Figure S1B,C). CFU analysis revealed that *S. enterica* Typhimurium 14028 Δ*tolRA* had a slightly longer doubling time than the wild-type *S. enterica* Typhimurium 14028 strain. The *S. enterica* Typhimurium 14028 Δ*tolRA* pBAT_*vioABCDE* strain also exhibited a longer doubling time than the *S. enterica* Typhimurium 14028 pBAT_*vioABCDE* strain.

To characterize the OMVs from the four strains, we analyzed OMVs isolated from LB cultures collected in the stationary phase (after 20 h of growth, OD_600_ ∼ 5) using Nanoparticle Tracking Analysis (NTA), total protein quantification by the Bradford method, Transmission electron microscopy (TEM), and High-performance liquid chromatography (HPLC) (Figure 1). As expected, our results showed that the deletion of the *tolR* and *tolA* genes significantly increased production (*p* = 0.0123) and protein concentration (*p* < 0.0001) of OMVs in *S. enterica* Typhimurium 14028 (Figure 1A,B). Additionally, we observed that transforming *S. enterica* Typhimurium 14028 Δ*tolRA* with pBAT_*vioABCDE* led to a significant decrease in OMVs production (*p* = 0.0285), likely due to the metabolic burden associated with violacein production in the mutant. The ST*vio*-OMV and *tolRAvio*-OMV samples appeared darker than ST-OMV and *tolRA*-OMV, indicating that the OMVs were loaded with violacein (Figure 1G).

**Figure 1:**
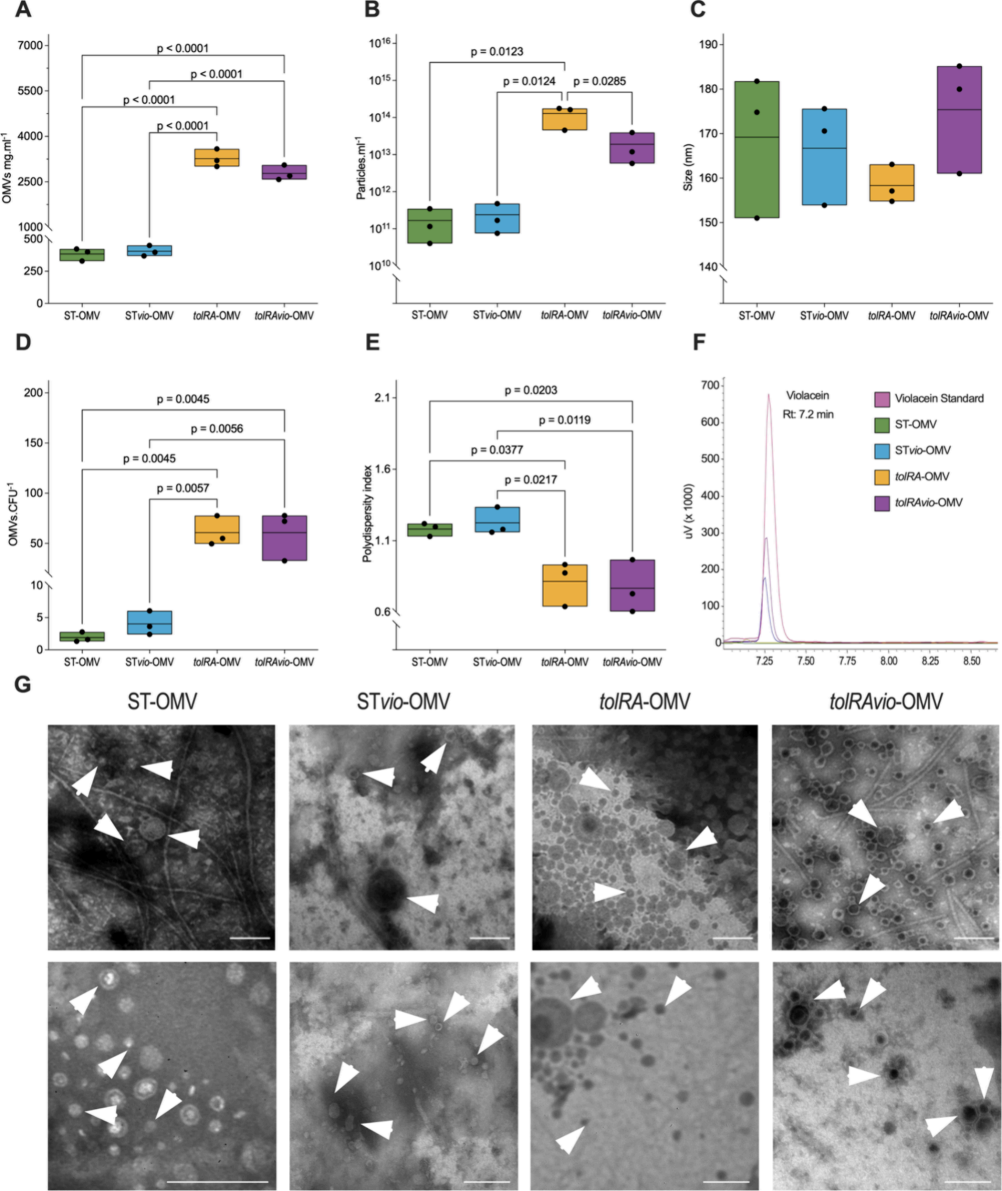
Characterization of OMVs isolated from *S. enterica* Typhimurium. OMVs were isolated from stationary phase cultures grown in the LB broth. (A) Total protein content in OMV samples. (B) OMVs quantified by nanoparticle tracking analysis (NTA). The deletion of the *tolR* and *tolA* genes in *S. enterica* Typhimurium 14028 results in an increased OMV production and total protein content. (C) Mean diameters of OMVs measured by NTA. (D) Number of OMVs produced per CFU. (E) OMV polydispersity index. Samples with a polydispersity index less than one are considered monodisperse, while those with an index greater than one are considered polydisperse. Data are in triplicate and were analyzed using one-way ANOVA. (F) Detection of violacein in OMVs by high-performance liquid chromatography (HPLC). (G) Transmission electron microscopy images of OMV. Representative OMVs are indicated by white arrowheads in the TEM images to assist with interpretation. Scale bars = 200 nm.

NTA measurements showed no significant differences in the mean diameters of OMVs isolated from the four strains (Figure 1C). The polydispersity index values for *tolRA*-OMV and *tolRAvio*-OMV were 0.81 and 0.76, respectively (Figure 1E), as estimated by NTA, suggesting a narrower size distribution compared to ST-OMV and ST*vio*-OMV, which exhibited higher polydispersity index values indicative of greater heterogeneity in OMVs size (Figure 1E and Figure S2). Our results also revealed a significantly higher number of OMVs normalized by CFU in samples from Δ*tolRA* mutants (*p* = 0.0045), compared to OMVs of *S. enterica* Typhimurium 14028 or *S. enterica* Typhimurium 14028 pBAT_*vioABCDE* (Figure 1D).

OMV preparations were evaluated by TEM with negative staining (Figure 1G). TEM visualization revealed similar bilayered, spherical particles across all four OMV preparations. Notably, dark staining was observed within ST*vio*-OMV and *tolRAvio*-OMV, indicating the presence of violacein in vesicles from strains transformed with pBAT_*vioABCDE*. TEM results confirmed violacein in vesicles from strains transformed with those measured by NTA. Specifically, ST-OMV ranged from approximately 57 to 220 nm in diameter, while ST*vio*-OMV ranged from 24 to 166 nm. The diameter of *tolRA*-OMV ranged from 35 to 149 nm, and for *tolRAvio*-OMV ranged from 27 to 93 nm, as determined by size measurements based on TEM images. Additionally, *tolRA*-OMV and *tolRAvio*-OMV exhibited lower size variability than ST-OMV and ST*vio*-OMV (Figure S2), aligning with the polydispersity index values obtained from the NTA data (Figure 1E). The greater heterogeneity observed in ST-OMV and STvio-OMVs, both in NTA and TEM, likely reflects their higher PDI and the intrinsic variability expected from nonhypervesiculated strains.^57^ Furthermore, TEM imaging of OMV preparations revealed the presence of numerous non-OMV structures, such as flagellin, a characteristic feature *Salmonella* OMVs.^57,58^

Previous studies have demonstrated that OMVs can carry hydrophobic compounds, and recent research has shown that OMVs isolated from *Chromobacterium violaceum* can transport violacein and facilitate its extracellular export.^37,59^ Based on these findings, we hypothesized that the purple coloration observed in ST*vio*-OMV and *tolRAvio*-OMV preparations, as well as the dark staining seen in TEM for these samples (Figure 1G), is due to the presence of violacein. We analyzed the OMV preparations for violacein content to test this hypothesis using HPLC (Figure 1F). Our analysis detected violacein in ST*vio*-OMV and *tolRAvio*-OMV but not in ST-OMV or *tolRA*-OMV. Furthermore, we found that *tolRAvio*-OMV contained four times more violacein than ST*vio*-OMV (Table S1).

### Violacein-Loaded OMVs Are Cytotoxic to MelanomaCells

To evaluate the antitumor efficacy of OMVs *in vitro*, we treated B16F10 cells with OMVs at various concentrations for 24 h and assessed cell viability using the MTT assay (Figure 2). Both SDS (Sodium dodecyl sulfate) and violacein controls were cytotoxic, with violacein having an IC_50_ of 1.55 μM (Figure 2A,F). In contrast, ST-OMV and *tolRA*-OMV were not cytotoxic at the tested concentrations (Figure 2B,D), indicating that empty vesicles are nontoxic to B16F10 cells. As anticipated, ST*vio*-OMV and *tolRAvio*-OMV exhibited cytotoxicity, with IC_50_ values of 4.28 × 10^9^ vesicles/mL and 9.30 × 10^8^ vesicles/mL, respectively. The IC_50_ of *tolRAvio*-OMV was significantly lower (*p* = 0.0008) than that of ST*vio*-OMV, likely due to the higher amount of violacein within *tolRAvio*-OMV that in ST*vio*-OMV, as demonstrated by HPLC (Figures 2C,E and 1F and Table S1). Based on HPLC data regarding the relative quantification of violacein in the vesicles, we found that the IC_50_ value was approximately 9-fold lower when violacein was encapsulated in OMVs compared to nonencapsulated violacein.

**Figure 2:**
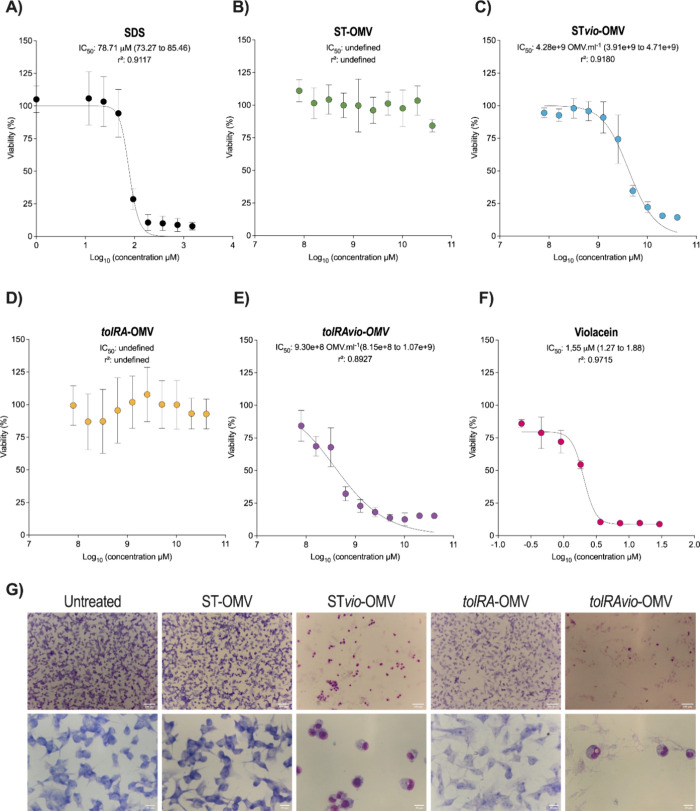
Cytotoxic profile of OMVs for B16F10 murine melanoma cells after 24 h of treatment. B16F10 murine melanoma cells were treated with serial dilutions of OMV, with the highest tested dilution: 2.036 × 10^10^ vesicles/mL. (A) Dose–response curve for SDS (sodium dodecyl sulfate, 150 μg/mL). (B) Cytotoxicity of ST-OMV. (C) Cytotoxicity of ST*vio*-OMV. (D) Cytotoxicity of *tolRA*-OMV. (E) Cytotoxicity of *tolRAvio-*OMV. (F) Cytotoxicity of violacein. Cell viability was measured using the MTT assay. Data are presented as mean ± SD from two independent experiments (*n* = 6), with values normalized to the untreated cell control. (G) Morphological changes in B16F10 cells treated with 5 × 10^9^ vesicles/mL of OMV. Scale bars: 100 μm (top images) and 20 μm (bottom images).

In addition to the MTT assay, the *in vitro* cytotoxicity of *tolRAvio*-OMV and ST*vio*-OMV was further assessed using microscopy and Giemsa staining. We observed decreased cell viability and notable morphological changes in cells treated with 5 × 10^9^ vesicles/mL of either *tolRAvio*-OMV or ST*vio*-OMV (Figure 2G). Given that no significant differences were found between the *in vitro* effects of ST-OMV and *tolRA*-OMV, combined with the superior yield of OMVs from the Δ*tolRA* mutant compared to those from *S. enterica* Typhimurium 14028 and the lower IC_50_ of *tolRAvio*-OMV compared to ST*vio*-OMV in melanoma cells, subsequent experiments focused exclusively on *tolRA*-OMV and *tolRAvio*-OMV.

The monolayer assay (2D culture) is limited and may overestimate cytotoxicity compared to a three-dimensional model (3D model).^60^ We assessed vesicle toxicity using a 3D model of B16F10 cells (Figure 3). Constructs in 96-well plates were treated with three concentrations of OMVs (2.0 × 10^10^, 1.0 × 10^10^, and 0.5 × 10^10^ OMV/mL). As expected, SDS treatment was cytotoxic, while violacein (ranging from 5.80 μM to 1.45 μM) did not significantly affect the viability of cell aggregates. OMVs without violacein and *tolRA*-OMV also showed no significant toxicity to B16F10 aggregates. Consistent with the monolayer assay results, *tolRAvio*-OMV exhibited significant toxicity (*p* < 0.0001), reducing cell viability by 14.4 to 63.7% in a dose-dependent manner (Figure 3).

**Figure 3:**
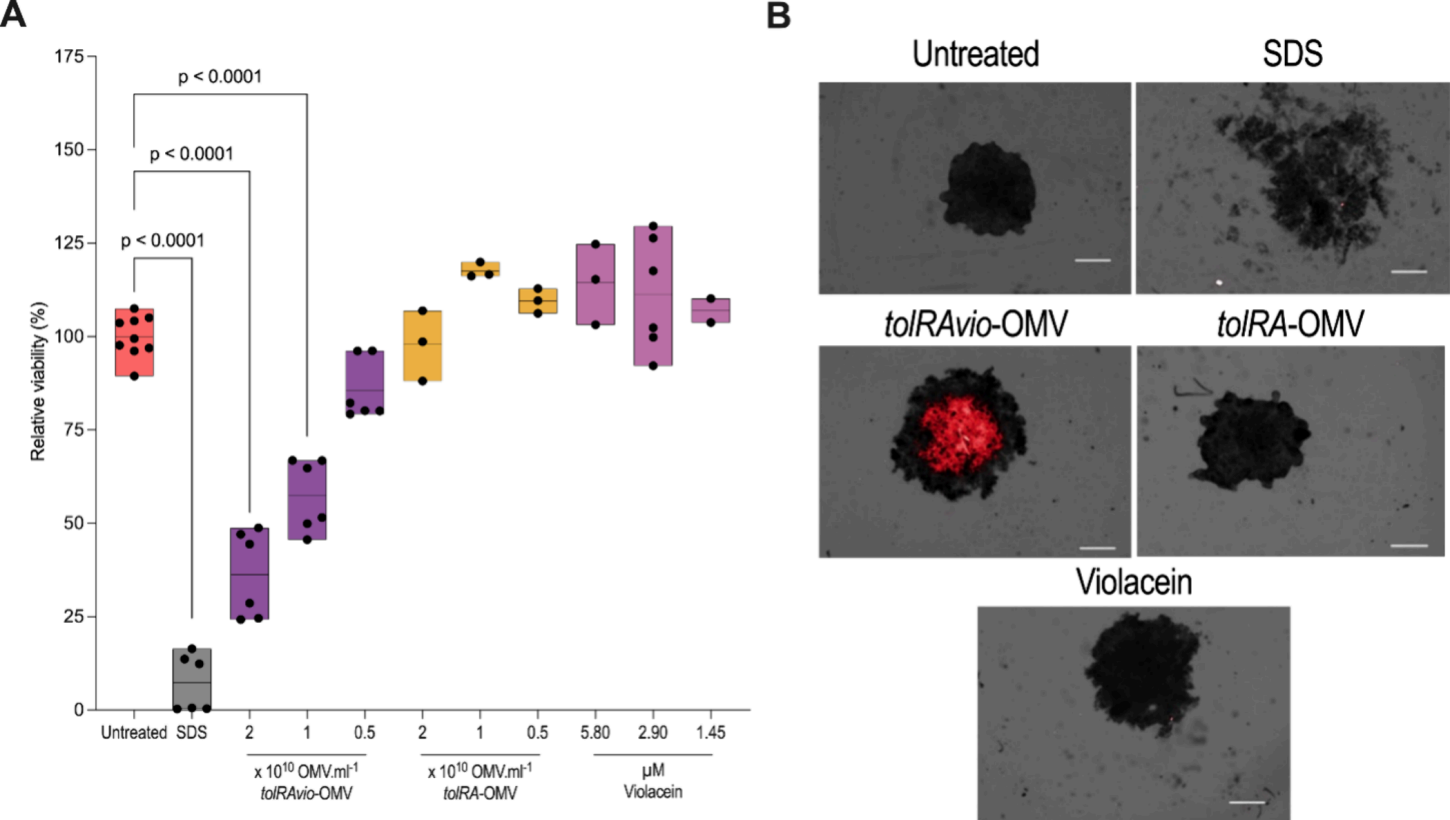
Three-dimensional model of B16F10 murine melanoma cells treated with OMVs for 24 h. (A) Untreated negative control (SDS, sodium dodecyl sulfate, 150 μg/mL) and positive death control. The OMV concentrations were (1) 2.036 × 10^10^ vesicles/mL, (2) 1.018 × 10^10^ vesicles/mL, and (3) 0.509 × 10^10^ vesicles/mL. For violacein, the concentrations were (1) 5.80 μM, (2) 2.90 μM, (3) 1.45 μM, and (4) 0.72 μM. Cell viability was assessed using the 3D CellTiter-Glo luminescent assay. Data are the results of two independent experiments with values normalized to the untreated control. Statistical significance was determined by one-way ANOVA. (B) Representative images of the three-dimensional B16F10 model. Cells (2 × 10^4^ per well) were seeded in a 96-well plate and treated with 2.036 × 10^10^ vesicles/mL for 24 h. Controls included cells without vesicles (untreated), cells treated with SDS (1500 μg/mL), and cells treated with violacein (5.08 μM). The red fluorescence signal corresponds to violacein autofluorescence (630 nm), which was used to monitor its cellular uptake. Red fluorescence was only observed in cells treated with *tolRAvio*-OMV, indicating the effective delivery of violacein into melanoma spheroids. *tolRAvio*-OMV exhibited significant toxicity in the 3D model. Images were acquired at 2× magnification using the High-Content Analysis Operetta 3.5. Scale bars: 50 μm.

### Concentration of Violacein in OMVs Correlates with Cytotoxic Effects

We also quantified the fluorescence intensity at 630 nm of *tolRAvio*-OMV and ST*vio*-OMV at a concentration of 2.75 × 10^9^ OMV/mL. Cells incubated with *tolRAvio*-OMV exhibited significantly higher fluorescence compared to those treated with ST*vio*-OMV (Figure S3). This suggests that *tolRAvio*-OMV delivers more violacein to the cells, possibly due to a higher concentration of violacein per vesicle as indicated by HPLC (Figure 1F), since both groups were tested at the same concentration of 2.75 × 10^9^ OMV/mL.

Additionally, we used 3D models to further assess the presence of violacein via fluorescence microscopy (Figure 3B). The red fluorescence observed in B16F10 melanoma cells corresponds to violacein, which exhibits intrinsic autofluorescence at approximately 630 nm. We leveraged this natural property to visualize the interaction between violacein-loaded OMVs and tumor cells. At a concentration of 2.75 × 10^9^ OMV/mL, *tolRAvio*-OMV-treated cells displayed clear red fluorescence localized around the nucleus, along with morphological changes, when compared to the control group. In contrast, treatment withST*vio*-OMV or free violacein did not produce detectable red fluorescence under the same imaging conditions, suggesting limited penetration of free violacein into the melanoma spheroids. This observation supports the hypothesis that OMV-based delivery enhances violacein uptake in a relevant 3D model. At a concentration of 2.04 × 10^10^ vesicles/mL, *tolRAvio*-OMV showed fluorescence at 630 nm and morphological changes in B16F10 cells compared to the control group (Figure 3B).

Estimates of the violacein content in *tolRAvio*-OMV and ST*vio*-OMV (by fluorescence at 630 nm) were consistent with the HPLC results (Table 1 and Table S1). By comparing the optical density of violacein-loaded OMVs to standard violacein concentrations, it was determined that *tolRAvio*-OMV contains three times more violacein than ST*vio*-OMV (Table 1). This supports our hypothesis that the higher violacein in *tolRAvio*-OMV correlates with increased cell death compared to ST*vio*-OMV.

**Table 1:** Estimated Concentration of Violacein in μM Based on the OMV Concentration per Milliliter As Determined from the Violacein Absorbance Standard Curve (*r* = 0.9987)

relative amount of violacein based on the absorbance curve		
OMV/mL	*tolRAvio*-OMV	ST*vio*-OMV
7.95 × 10^7^	0.4292	0.1392
1.59 × 10^8^	0.8580	0.2785
3.18 × 10^8^	1.7161	0.5569
6.36 × 10^8^	3.4324	1.1138
1.27 × 10^9^	6.8539	2.2242
2.55 × 10^9^	13.7619	4.4660
5.09 × 10^9^	27.4698	8.9145
1.02 × 10^10^	55.0476	17.8641
2.04 × 10^10^	110.0952	35.7282
4.08 × 10^10^	220.1904	71.4565
8.16 × 10^10^	440.3807	142.9130

In summary, our *in vitro* data suggest that *tolRAvio*-OMV is four times more cytotoxic than ST*vio*-OMV, attributed to its high internal concentration of violacein, given that the number of OMVs was similar. Conversely, ST-OMV and *tolRA*-OMV did not exhibit cytotoxic effects *in vitro*. *tolRAvio*-OMV contains three to four times more violacein than ST*vio*-OMV, as confirmed by both absorbance measurements and HPLC (Figure 1F and Figure S3). Compared to free violacein, *tolRAvio*-OMV achieves the IC_50_ concentration with nine times less violacein (Figure 2C,E,F). Thus, we conclude that using OMV promotes a better biological efficacy of violacein treatment.

### OMVs Deliver Violacein into Tumor Cells

Based on the HPLC results, we investigated whether OMVs could effectively deliver violacein to melanoma cells. B16F10 cells were treated with various concentrations of OMVs (Figure 4A). When cells treated with *tolRAvio*-OMV were excited by light at 570 nm, violacein was detected as red fluorescence in the cytoplasm, indicating successful delivery of violacein. Fluorescence was observed only in cells treated with *tolRAvio*-OMV at a concentration of 2.75 × 10^9^ OMV/mL and not in those treated with ST*vio*-OMV. This concentration was selected based on the cytotoxic profile of OMVs experiments to identify a physiologically relevant dose that produced measurable violacein delivery and cytotoxic effects, while avoiding complete loss of cell viability. Even at the highest concentration (1.02 × 10^10^ OMV/mL), ST-OMV and *tolRA*-OMV did not exhibit cytotoxicity or red fluorescence since they do not contain violacein (Figure 4A). Additionally, analysis of morphology in cells treated with *tolRAvio*-OMV at 2.75 × 10^9^ OMV/mL revealed vacuoles (black arrows) within the B16F10 cells, alongside the violacein fluorescence (red fluorescence) (Figure 4B,C).

**Figure 4:**
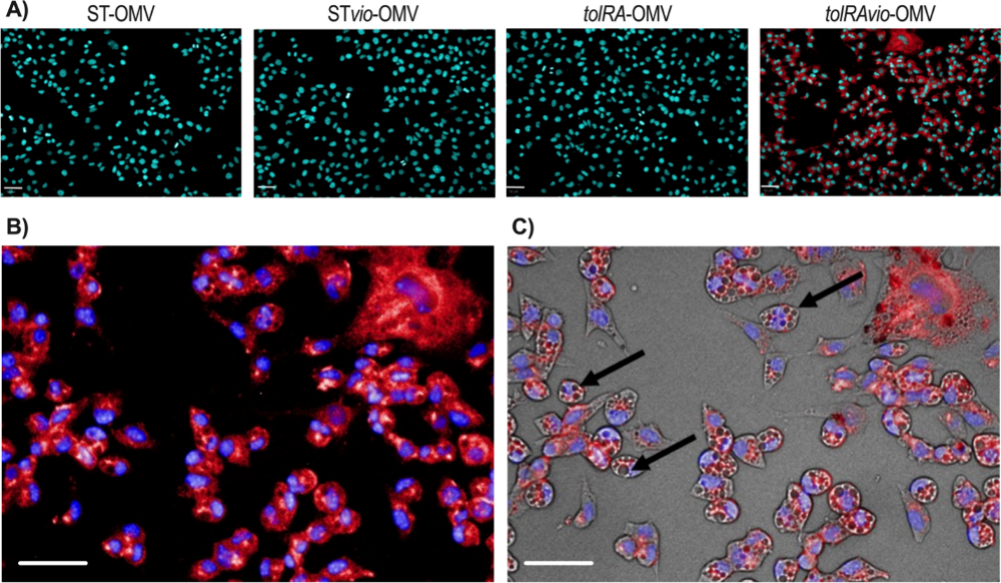
Representative fluorescence microscopy images of B16F10 cells. (A) Cells were seeded in 96-well microplates at a density of 1.0 × 10^4^ eukaryotic cells per well and treated for 4 h with 1.02 × 10^10^ vesicles/mL of ST-OMV and *tolRA*-OMV or 2.75 × 10^9^ vesicles/mL of *tolRAvio*-OMV and ST*vio*-OMV. (B, C) B16F10 cells were treated with 2.75 × 10^9^ particles/mL of *tolRAvio*-OMV for 24 h. Images show bright-field (B) and dark-field (C) views, with arrows indicating vacuolation. Nuclei were stained with DAPI (blue, ex: ∼350/470 nm), and violacein was stained red (ex: ∼575/675 nm). Images were acquired at 20× magnification using the High-Content Analysis Operetta 3.5. Scale bars: 50 μm.

### Violacein-Loaded OMVs Demonstrate Safety in Mice

For the tumor treatment in mice, we evaluated the toxicity of three doses (5 × 10^9^, 1 × 10^10^ and 2 × 10^10^ vesicles) of *tolRA*-OMV and *tolRAvio*-OMV, administered subcutaneously twice a week for 2 weeks, totaling four doses (Figure 5A). After treatment of mice with the first and second doses (first week), we observed weight loss in both with *tolRA*-OMV (15%) and with *tolRAvio*-OMV (13%) (Figure 5C,D). No weight loss was noted in the PBS control group (Figure 5C,D). Additionally, signs of illness, such as piloerection and lethargy, were observed in the groups receiving the highest concentrations of *tolRA*-OMV and *tolRAvio*-OMV during the first 7 days following the start of treatment (2 × 10^10^ vesicles). However, the groups 5 × 10^9^ *tolRA*-OMV and *tolRAvio*-OMV did not show any clinical signs of systemic toxicity beyond the weight loss.

**Figure 5:**
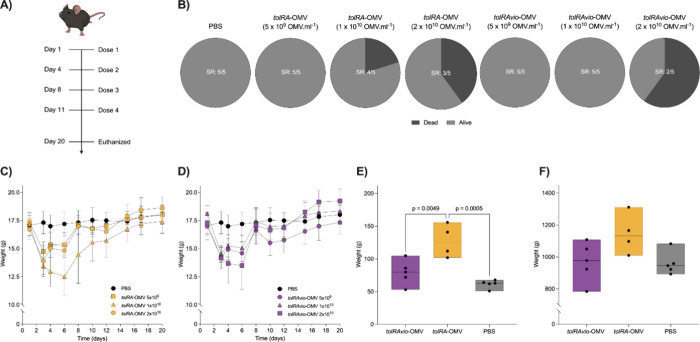
Evaluation of treatment safety with different doses of OMVs in healthy mice. (A) Experimental design for assessing the toxicity of various doses of OMVs in subcutaneously inoculated mice. (B) Survival rate (SR) of mice inoculated with different doses of OMVs at the study end point. (C) Body weight changes of mice inoculated with different doses of *tolRA*-OMV over the testing period. (D) Body weight changes of mice inoculated with different doses of *tolRAvio*-OMV over the testing period. (E) Spleen weights of mice inoculated with 5 × 10^9^ OMVs. (F) Liver weights of mice inoculated with 5 × 10^9^ OMVs at the end point of the experiment. The experiment was conducted twice independently with similar results. Statistical significance was assessed using one-way ANOVA.

One week after starting the inoculations, the mice recovered from the initial clinical symptoms caused by high concentrations of *tolRA*-OMV and *tolRAvio*-OMV and began to regain weight. By the end of the experiment, there were no significant differences in weight between the PBS control group and those treated with *tolRA*-OMV or *tolRAvio*-OMV. Additionally, no mice treated with 5 × 10^9^ *tolRA*-OMV or *tolRAvio*-OMV had died by the end of the experiment (Figure 5B).

Macroscopic examination of the spleen revealed splenomegaly in the 5 × 10^9^ *tolRA*-OMV group, with significant differences in spleen weight compared to the control group and the 5 × 10^9^ *tolRAvio*-OMV group (*p* = 0.0005 and *p* = 0.0049, respectively) (Figure 5E). Although we observed increased liver weight in the 5 × 10^9^ *tolRAvio*-OMV group, the differences were not statistically significant (Figure 5F). Splenomegaly was also noted in mice inoculated with the highest concentrations of either *tolRA*-OMV or *tolRAvio*-OMV (Figure S4).

We also performed a histological examination of the main organs in mice inoculated with 5 × 10^9^ vesicles to assess the potential toxic effects of OMVs on this dose (Figure S5). Microscopic analysis of spleen, liver, kidney, and lung sections from control groups revealed typical organ architecture. In contrast, mice treated with *tolRA*-OMV and *tolRAvio*-OMV showed mild histological changes in the spleen and liver. Specifically, the spleens of these mice exhibited hypertrophy in both the white and red pulp, along with an increased number of megakaryocytes in the red pulp compared to the control group. In the liver, both *tolRA*-OMV and *tolRAvio*-OMV treatments resulted in mild inflammatory cell infiltration (polymorphonuclear cells and macrophages) around the centrilobular veins without evidence of hepatocyte degeneration.

### Both Violacein-Loaded and Unloaded OMVs Reduce Tumor Growth in Mice

The safety evaluation of the treatments indicated that the mice tolerated a dose of 5 × 10^9^ OMV well. Therefore, this dose was selected for the antitumor efficacy experiments. We assessed the ability of *tolRA*-OMV and *tolRAvio*-OMV to inhibit tumor growth in a murine melanoma model. By quantifying the violacein content in the vesicles using HPLC, we determined that 5 × 10^9^ *tolRAvio*-OMV contains approximately 306 ng of violacein. To further investigate the effects of the violacein present in the vesicles, tumor cells were injected subcutaneously into the right flank of C57BL/6JUnib mice. Approximately 10 days after tumor cell inoculation, tumors reached ∼100 mm^3^, and mice were randomized into four groups to receive intratumoral injections of PBS, 306 ng violacein, 5 × 10^9^ *tolRA*-OMV, or 5 × 10^9^ *tolRAvio*-OMV.

We observed significant inhibition of tumor growth in mice treated with either *tolRA*-OMV or *tolRAvio*-OMV compared to the PBS control group (*p* < 0.0001) (Figure 6). Six days after the first dose of *tolRA*-OMV or *tolRAvio*-OMV, tumors in most mice were no longer visible. Notably, *tolRAvio*-OMV treatment resulted in a faster reduction in tumor size than *tolRA*-OMV. Pure violacein treatment also slowed tumor growth but was less effective (Figure 6). By the end of the experiment, tumors were absent in all mice treated with *tolRAvio*-OMV. In the *tolRA*-OMV group, although tumors were not observed in 5 out of 7 mice, in 2 mice, the tumors were visible, contrasting with the results obtained with *tolRAvio*-OMV treatment. These results indicate the superior efficacy of OMV-carrying violacein. In contrast, pure violacein treatment did not promote a significant difference in tumor size compared to PBS treatment (Figure 6C).

**Figure 6:**
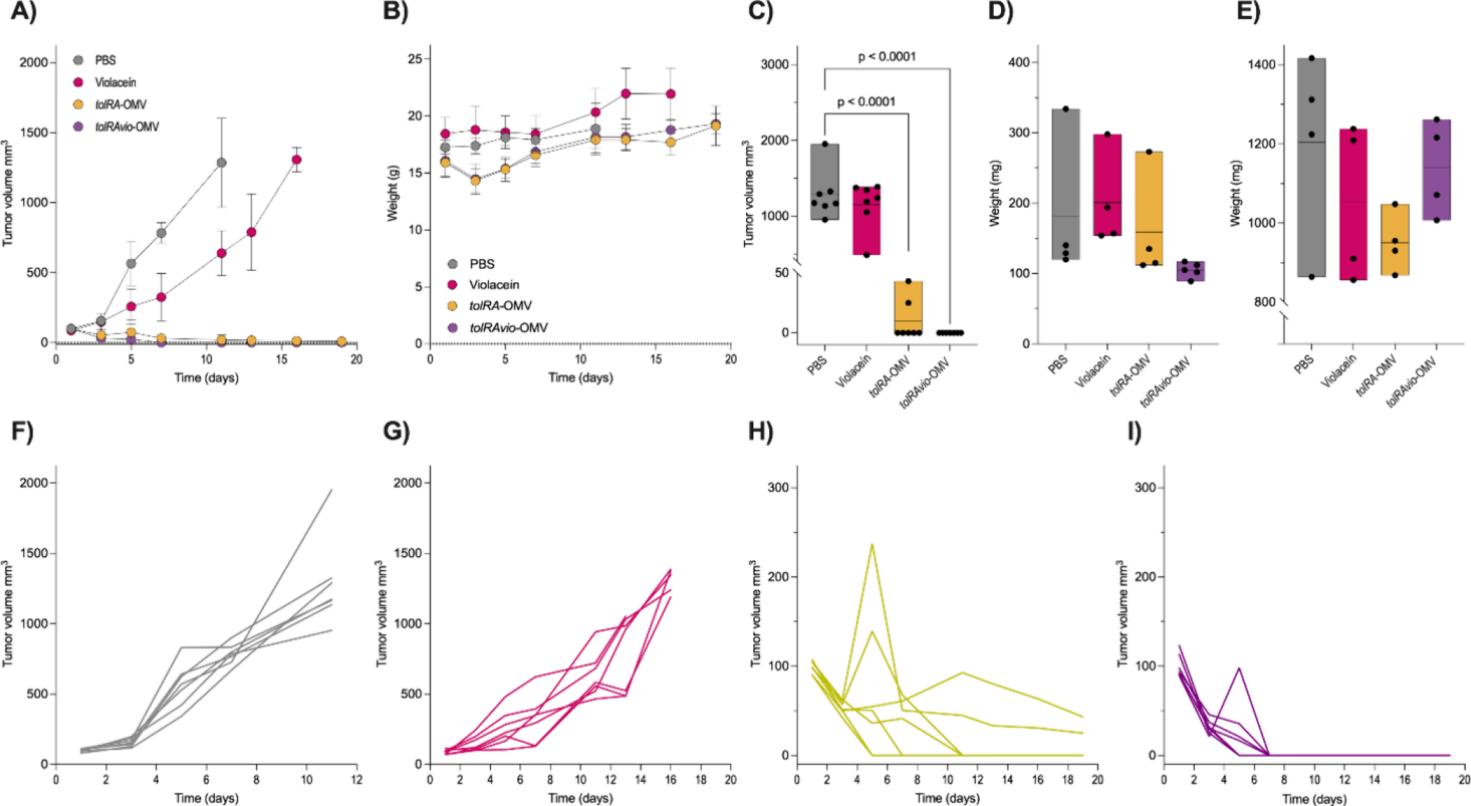
Antitumor efficacy of OMVs in a murine melanoma model. Tumor growth suppression was evaluated in a B16F10 melanoma model using C57BL/6JUnib mice. Tumor-bearing mice (∼100 mm^3^) were treated intratumorally twice a week for 2 weeks with PBS, *tolRA*-OMV (5 × 10^9^ vesicles), *tolRAvio*-OMV (5 × 10^9^ vesicles), or violacein (306 ng). (A) Tumor growth during the treatment period. (B) Body weight of mice during the treatment period. (C) Tumor size at the experiment end point. (D) Spleen weight and (E) liver weight following treatments. Individual tumor growth kinetics of mice treated with PBS (F), violacein (306 ng) (G), *tolRA*-OMV (5 × 10^9^ vesicles) (H), or *tolRAvio*-OMV (5 × 10^9^ vesicles) (I) are shown. The experiment was conducted twice independently with similar results. Statistical significance was assessed by one-way ANOVA.

We also observed that treatments with OMVs did not cause lethality in mice. However, mice treated with violacein and PBS had to be euthanized before the experiment’s end point to prevent further suffering due to exacerbated tumor growth. Regarding weight, there was a 10% decrease in body weight after the first dose of *tolRA*-OMV and *tolRAvio*-OMV; however, mice regained their weight within a week (Figure 6B). By the end of the experiment, there were no significant differences in the weights of the different groups. Additionally, no significant differences in spleen or liver size were observed between the groups (Figure 6D,E).

H&E staining of tumor sections from mice treated with PBS showed destruction of the epidermis, necrotic regions, vascular proliferation, and a high presence of invading neoplastic cells. In contrast, tumors from the violacein-treated group exhibited some apoptotic cells, invasive neoplastic cells, and continued vascular proliferation. Tumors from the *tolRA*-OMV or *tolRAvio*-OMV groups, however, displayed extensive necrosis, a high number of apoptotic cells, stromal fibrosis, and a moderate inflammatory infiltrate, including polymorphonuclear cells, lymphocytes, and macrophages (Figure S6).

Microscopic examination of the spleens, livers, lungs, and kidneys did not reveal significant tissue damage in mice treated with violacein, *tolRA*-OMV, or *tolRAvio*-OMV compared to the control group (Figures S5, S7). We observed moderate inflammatory infiltration in the spleens, livers, and lungs of the mice treated with *tolRA*-OMV or *tolRAvio*-OMV, and mild inflammatory infiltration in those treated with violacein. Additionally, there was an increase in Kupffer cells in the livers of mice treated with violacein, *tolRA*-OMV, or *tolRAvio*-OMV. Liver tissues from *tolRAvio*-OMV-treated mice also exhibited mild degenerative changes in hepatocytes. Given tumor growth inhibition, survival rates, and minimal adverse effects on organs, these results suggest that *tolRAvio*-OMV treatment offers the most effective therapeutic outcome with minimal side effects and 100% survival.

### OMVs Trigger Tumor Regression through an Inflammatory Response

We also investigated whether OMV treatments could stimulate an antitumor immune response in mice. The antitumor efficacy results indicated that *tolRAvio*-OMV significantly reduced tumors within approximately 6 days. To analyze the immune response in tumor tissue before regression, we administered two doses of OMVs to tumor-bearing mice, with a four-day interval between doses. The mice were euthanized four hours after the second dose, and tumor tissues were collected for immune response analysis.

We assessed the macrophages population and their phenotype in the tumor tissue using flow cytometry with antibodies against CD80-Pe, CD206-APC, CD11b-Percp, and F4/80-Fitc (Figure 7A). Additionally, we evaluated the expression of genes associated with the antitumor response by qRT-PCR. Our data showed that treatment with *tolRA*-OMV and *tolRAvio*-OMV significantly increased the accumulation of macrophages (CD11b+_F4/80+ cells) in the tumor tissue compared to mice treated with PBS (*p =* 0.0055 and *p =* 0.0324 respectively) (Figure 7B).

**Figure 7:**
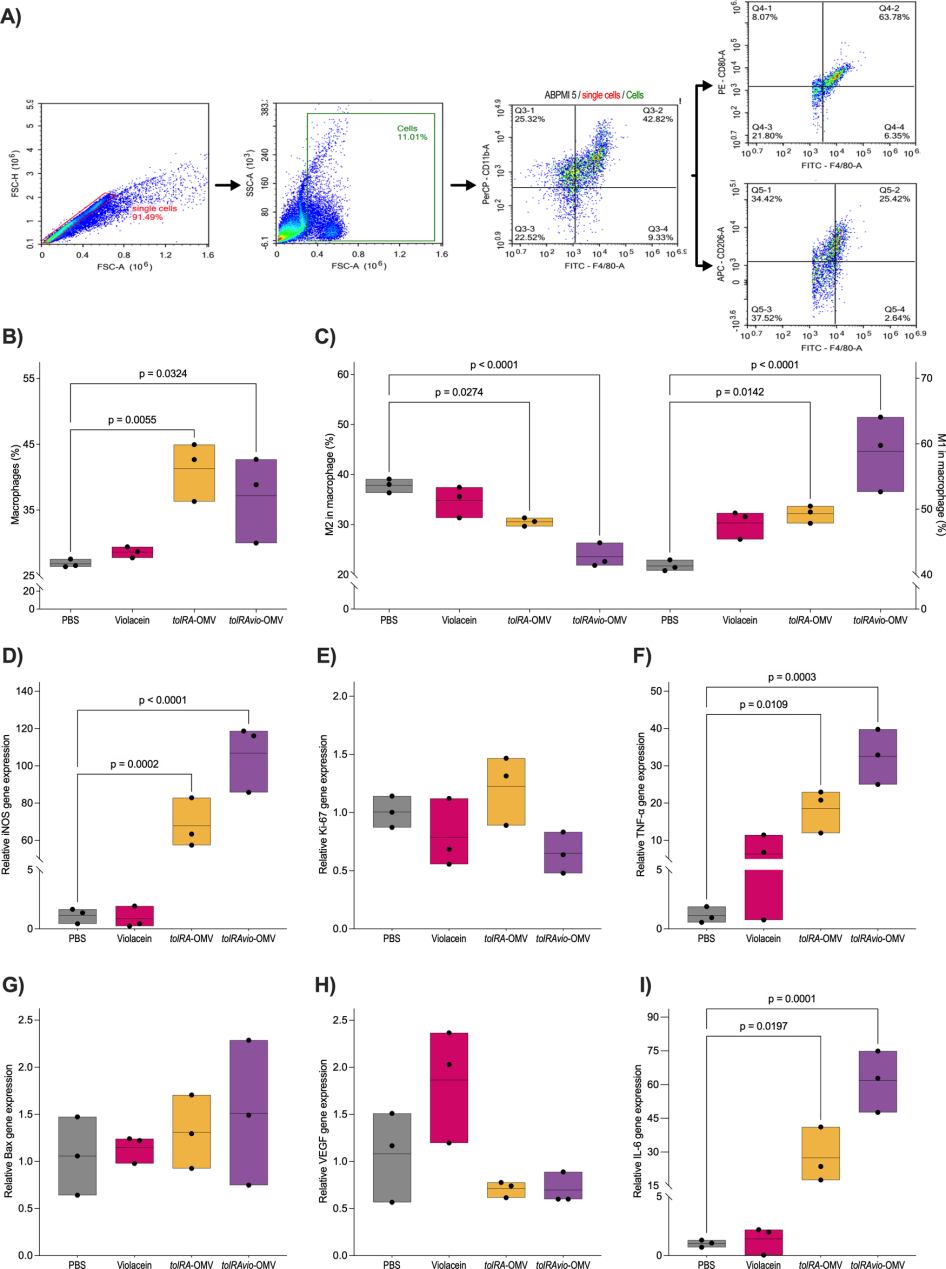
Mediators and macrophages are involved in the antitumor effect of OMV. (A) Representative gating strategy to detect the total macrophage (F4/80+ _CD11b+), M1 phenotype macrophages (F4/80+ _CD80+), and M2 phenotype macrophages (F4/80+ _CD206+) in the tumor tissue from mice treated with OMV. (B) Percentage of total macrophages and their phenotypes. (C) Immunophenotyping of M1 and M2 macrophages. (D–I) Relative mRNA expression levels of the following genes: iNOS (D), *K*_i_-67 (E), TNF-α (F), Bax (G), VEGF (H), and IL-6 (I). The tumor tissue from mice bearing B16F10 tumors was collected 4 h after the second intratumoral inoculation of OMV, violacein, or PBS. Data are related to three mice per group. Statistical significance was determined by one-way ANOVA.

We found no significant differences in the percentage of macrophages between the PBS group and the violacein treatment (Figure 7B). Next, we investigated the phenotype of macrophages in tumor tissue (Figure 7C). To differentiate between M1 and M2 macrophages, we analyzed the expression of CD80 and CD206 in F4/80+ cells. The population of M1 macrophages (F4/80+_CD80+) in tumors treated with *tolRA*-OMV and *tolRAvio*-OMV increased significantly (*p* = 0.0142 and *p* < 0.0001, respectively) compared to the PBS-treated group. In contrast, the population of type M2 macrophages (F4/80+_CD206+) decreased significantly (*p* = 0.0274 and *p* < 0.0001, respectively; Figure 7C) in tumors treated with *tolRA*-OMV and *tolRAvio*-OMV when compared to the group treated with PBS. In addition, the induction of M1 and decrease of M2 macrophages were observed in mice inoculated with the *tolRAvio*-OMV compared to the *tolRA*-OMV group (Figure 7C). These results indicate that OMV from *S. enterica* Typhimurium induces tumor suppression by promoting the accumulation of M1-type macrophages in the tumor tissue, but that this response is further promoted by violacein.

Some cytokines secreted by immune cells can directly destroy tumor cells or enhance antitumor responses.^61^ Therefore, we analyzed the mRNA expression of several inflammatory mediators in tumor tissue (Figure 7). The results revealed that the expression levels of iNOS, TNF-α, and IL-6 mRNA were significantly higher in tumors treated *tolRA*-OMV and *tolRAvio*-OMV compared to those treated with PBS or violacein. Notably, tumors treated with *tolRAvio*-OMV exhibited the highest levels of these transcripts (Figure 7D,F,I), suggesting that violacein delivered by vesicles enhances immune activation and therapeutic efficacy, particularly related to iNOS and IL-6 levels (*p* = 0.0125 and *p* = 0.0078, respectively) and showing a trend toward increased TNF-α (*p* = 0.0531), when *tolRA*-OMV and *tolRAvio*-OMV were compared (data not shown). Additionally, we observed higher expression of the Bax transcript and a lower expression of VEGF in tumors treated with *tolRA*-OMV and *tolRAvio*-OMV. However, these differences were not statistically significant.

## Discussion

The antitumor efficacy of violacein has been well-documented.^19,25^ However, its clinical application has been limited due to its high hydrophobicity and nonspecific toxicity.^12,28^ Previous studies have shown that tumor cells are more sensitive to violacein than nontumor cells. Using violacein delivery systems could reduce its required therapeutic concentration and minimize nonspecific toxicity.^21^

Strategies to enhance the delivery and reduce the toxicity of violacein have primarily focused on polymeric nanoparticle systems, emulsified microspheres, and other carrier-based approaches.^28,62–64^ While these strategies have demonstrated high stability, they have not been very effective in improving the delivery of violacein to the target cells. Furthermore, the uniform large-scale production of such nanoparticles remains complex and costly.^65^ A novel strategy involving the administration of violacein through *J. lividum* extracellular vesicles has recently been explored *in vitro*. Notably, studies in tumor cells demonstrated that violacein encapsulated in these vesicles retained its antitumor activity, highlighting its potential as a biocompatible delivery platform.^45^

In contrast, our study advances this field by evaluating violacein-loaded OMVs *in vitro* (2D and 3D culture) and *in vivo*, specifically in a murine melanoma model. Furthermore, we utilized hypervesiculated mutants of *S. enterica* Typhimurium to enhance OMV production yields, a critical advantage for scalable manufacturing and clinical translation. These engineered OMVs facilitated efficient violacein entry into tumor cells and triggered immune responses that synergistically eliminated melanoma tumors. This dual approach addresses key limitations of traditional nanoparticle systems and positions bacterial OMVs as viable platforms for anticancer drug delivery.

The use of OMV, alone or as nanocarriers, for developing new therapies has surged in recent years.^44,66–69^ The stability, cost-effectiveness, and success of OMV-based vaccines, such as those for meningitis, highlight their potential for developing efficient treatments. However, one challenge has been the low yield of OMVs from wild-type bacterial cultures. To address this, we employed a strain of *S. enterica* Typhimurium lacking the TolA and TolR proteins. Our findings align with other studies that have demonstrated a significant increase in OMV yield from mutants deficient in these proteins compared to wild-type strains (Figure 1).^66,69,70^

Notably, OMVs derived from the Δ*tolRA* mutant exhibited a narrower size distribution compared to wild-type OMVs (Figure 1E and Figure S2).^71–73^ This is a desirable feature for biomedical applications, as a high degree of uniformity in nanoparticle populations typically enhances reproducibility, bioavailability, and safety.^71–73^ The polydispersity index observed for wild-type *Salmonella* OMVs was relatively high. However, OMVs isolated from the Δ*tolRA* mutant strain displayed a lower PDI, more consistent with values reported for OMVs from other bacterial species such as *Brucella abortus*, *Neisseria lactamica* and *Acinetobacter baumannii*, supporting their suitability as delivery platforms.^74–77^ Overall, this level of polydispersity is standard in naturally derived OMVs due to their biological origin and the complexity of their biogenesis.^35^ Nevertheless, the improved size distribution achieved using the Δ*tolRA* strain is encouraging for translational applications.

The presence of non-OMV structures (Figure 1G), including flagellin, in our OMV preparations is consistent with previously reported findings and highlights the inherent structural diversity of vesicle production across bacterial species.^57,58,78^ In *S. enterica*, this diversity is particularly notable due to the frequent presence of flagellin, likely reflecting intrinsic bacterial properties and vesicle biogenesis pathways.^36^ While such complexity offers a more representative view of native OMV populations, further downstream purification or genetic strategies, such as flagellin knockouts, could be employed to enhance OMV purity for applications that require greater compositional consistency.^78^ As a foundational study, we prioritized establishing a proof of concept for the potential antitumor role of violacein-loaded OMVs. We acknowledge that additional work will be needed to optimize OMV purification and uniformity to fully support future translational applications.

This study primarily focused on evaluating the antitumor efficacy of violacein-loaded OMVs; however, the scalability of their production represents a critical aspect of clinical translation. *S. enterica* Typhimurium Δ*tolRA* strain used in this work displays a hypervesiculating phenotype, which significantly enhances OMV yield and homogeneity compared to wild-type strains (Figure 1A,B), a key advancement for large-scale manufacturing. Extensively implemented for attenuated pathogenic bacteria, bioreactor cultivation methods could optimize OMV production. The intrinsic loading of violacein during bacterial synthesis eliminates additional encapsulation steps, streamlining the process and reducing costs relative to synthetic systems. Although OMVs retained functionality when stored at −80 °C for up to 6 weeks, future studies will systematically evaluate long-term stability to define optimal storage conditions and ensure reproducibility.

Toxicity remains a significant challenge in cancer therapies that use bacteria or bacterial products. OMVs isolated from attenuated strains of *S. enterica* Typhimurium offer a safer alternative for cancer treatment. In this study, we utilized an attenuated, hypervesiculated, violacein-producing strain to isolate violacein-loaded OMV. Previously, we demonstrated the attenuation of this strain’s virulence *in vivo*.^50^ Our *in vitro* studies revealed that OMVs alone (ST-OMV and *tolRA*-OMV) did not reduce tumor cell viability, as shown by the MTT assay and 3D culture microscopy (Figures 2 and 3). In contrast, violacein-loaded OMVs (ST*vio*-OMV and *tolRAvio*-OMV) exhibited significant cytotoxicity against tumor cells.

Monolayer assay (2D culture) is limited and may overestimate cytotoxicity compared to a three-dimensional model.^60^ In 2D and 3D culture assays, we observed significant differences in the toxicity of ST*vio*-OMV and *tolRAvio*-OMV. To understand the underlying cause, we quantified the concentration of violacein in each OMV preparation. Relative quantification using HPLC and absorbance analysis revealed that *tolRAvio*-OMV contains three to four times more violacein than ST*vio*-OMV (Figure 1F, Figure S3, Table 1, and Table S1). This suggests that the enhanced toxicity of *tolRAvio*-OMV *in vitro* is attributable to its higher violacein content. Notably, the IC_50_ value for violacein delivered in OMVs (*tolRAvio*-OMV) was reduced by 9-fold compared to pure violacein. This is consistent with studies showing a 15-fold decrease in IC_50_ when violacein is encapsulated in polymeric nanoparticles.^79^ These findings indicate that delivering violacein via OMVs significantly enhances its anticancer efficacy at lower concentrations.

OMVs can adhere to and fuse with eukaryotic cells.^80–83^ Leveraging the autofluorescence of violacein, we visualized its presence within cells using fluorescence microscopy. Our data indicated that OMVs carrying violacein are taken up by melanoma cells, as evidenced by the red fluorescence observed around the nucleus (consistent with the presence of violacein) (Figure 4). Specifically, when cells were treated with 2.75 × 10^9^ vesicles/mL of *tolRAvio*-OMV (containing 0.47 μM of violacein), we observed intense fluorescence. In contrast, cells treated with ST*vio*-OMV (containing 0.12 μM of violacein) did not show noticeable fluorescence (Figure 4). These findings suggest that OMVs enhance the delivery of the hydrophobic compound, leading to more efficient cytotoxic effects, as demonstrated by the MTT or ATP assays. The precise mechanism underlying this process of adhesion and fusion of OMVs with eukaryotic cells warrants further investigation, which should be explored in future experiments.

The vacuolization observed in melanoma cells (B16F10) treated with *tolRAvio*-OMV in our study aligns with recent findings in melanoma cells exposed to violacein-loaded outer membrane vesicles (OMVs) isolated from *J. lividum*.^45^ Similarly, cytoplasmic vacuole formation in cells treated with violacein has been previously documented.^84^ Prior studies propose that this vacuolization may arise from intracellular alterations triggered during the early stages of programmed cell death. However, the relationship between vacuole formation, the compound’s bioactivity, and associated cellular signaling pathways remains incomplete, highlighting the need for further mechanistic studies to elucidate these interactions.

Given that *tolRAvio*-OMV demonstrated superior antitumor activity compared to ST*vio*-OMV and that the yield of OMVs from the Δ*tolRA* mutant was significantly higher than from the wild-type strain, we focused our *in vivo* experiments on *tolRAvio*-OMV. To evaluate systemic tolerability, we tested three escalating doses of *tolRA*-OMV and *tolRAvio*-OMV in mice. The two highest doses induced transient weight loss, clinical signs of distress (piloerection, lethargy), and mortality during the first week. In contrast, the lowest dose (5 × 10^9^ vesicles) caused only mild, reversible effects without mortality or significant weight loss (Figure 5). Histological analysis revealed preserved organ architecture, with mild spleen and liver alterations consistent with immune activation (Figure S5). Based on these results, the lowest dose was selected for subsequent efficacy studies. Although these findings support initial tolerability, future studies should incorporate detailed toxicological assessments such as cytokine profiling, blood chemistry, and liver enzyme analysis and explore less toxic OMVs to improve systemic safety.^85^

Bacterial and mammalian cell-derived vesicles (exosomes) have been recognized as effective drug delivery systems in cancer therapy.^39,44,86,87^ However, bacteria offer significant advantages over exosomes, including the ability to be cultured on a large scale more efficiently and cost-effectively.^65,88^ Additionally, bacterial vesicles retain a chemical composition similar to their parent bacteria on their surface, which may enhance their potential for cancer immunotherapy.^89^

Our antitumor experiments in a murine melanoma model demonstrated the efficacy of violacein-loaded OMV. These OMVs successfully eliminated tumors without causing lethality. As observed in other studies, violacein treatment reduced tumor growth compared to the control group but was less effective in eradicating the tumor (Figure 6).^15,25,26^ It is important to note that the amount of violacein present in 5 × 10^9^ vesicles (306 ng) is significantly lower than that typically used in subcutaneous tumor models, ranging from 13,000 to 27,000 ng.^15,26^ This suggests that OMVs reduce the required dosage of violacein and enhance its antitumor activity.

In contrast to the *in vitro* results, where OMVs without violacein did not exhibit antitumor activity (Figures 2, 4), significant antitumor effects were observed *in vivo* (Figure 6). Other studies have reported the antitumor activity of OMVs alone from *S. enterica* Typhimurium, such as in the treatment of solid Ehrlich Carcinoma in mice.^33^ This suggests that, like live bacteria, OMVs can activate the immune system due to their composition, which includes molecular patterns associated with pathogens from their parent bacteria.^44,89,90^ OMVs can interact with antigen-presenting cells and macrophages in the tumor microenvironment, triggering signaling pathways that recruit immune cells and promote the production of pro-inflammatory mediators.^91,92^ The flow cytometry analysis showed a significantly higher percentage of tumor-infiltrating macrophages in mice treated with *tolRA*-OMV and *tolRAvio*-OMV compared to control mice treated with PBS (Figure 7B). These results imply that OMVs from *S. enterica* Typhimurium can induce macrophage-mediated antitumor responses, similar to those elicited by live bacteria.

Tumors, particularly aggressive ones like melanoma, often develop an immunosuppressive microenvironment, contributing to their resistance and progression.^93–95^ This environment is characterized by an abundance of regulatory T cells and tumor-associated macrophages of the M2 phenotype, which facilitate immune evasion and tumor growth.^92^ A higher presence of M2-type macrophages has been correlated with a worse prognosis in melanoma.^94,96–98^ Effective cancer therapies must target tumor cell death in the tumor microenvironment to enhance treatment outcomes.^99^ Reprogramming the tumor microenvironment is, therefore, a promising strategy.

Flow cytometry analysis revealed a significantly higher ratio of M1-type to M2-type macrophages (M1/M2 > 1) in mice treated with *tolRA*-OMV or *tolRAvio*-OMV. In contrast, a lower ratio was observed in mice treated with PBS and violacein (Figure 7C). This suggests that OMVs may induce the polarization of macrophages from the M2 to the M1 phenotype. These findings align with previous studies indicating that *E. coli* OMVs can alter the tumor microenvironment by shifting macrophage polarization from the M2 to M1 phenotype.^92^ M1 macrophages are known for their pro-inflammatory properties and ability to produce cytokines that suppress tumors, contrasting with the anti-inflammatory and tumor-promoting role of M2 macrophages.^100–102^

The observed shift from M2 to M1 macrophages in our study was accompanied by a significant increase in the mRNA levels of iNOS, TNF-α, and IL-6 in tumor tissues (Figure 7C,D,F,I). Previous *in vitro* research has shown that violacein treatment increases the secretion of IL-6 and TNF-α in murine macrophages, and similar increases in these cytokines have been associated with an enhanced antitumor immune response.^27,39,92,103^ M1Macrophages are characterized by their nitric oxide production, and iNOS expression is a hallmark of this macrophage phenotype. Nitric oxide inhibits tumor cell proliferation by disrupting DNA synthesis.^104,105^ Our results indicate that the elevated iNOS levels likely contribute to reprogramming the tumor microenvironment. Notably, violacein-loaded OMVs induced a higher proportion of M1 macrophages and elevated expression of pro-inflammatory cytokines than OMVs without violacein. This suggests that violacein enhances the antitumor efficacy of OMVs by promoting a more robust M1 macrophage response and increasing pro-inflammatory mediator levels.

## Conclusions

Our study demonstrates that OMV derived from hypervesiculated mutant *S. enterica* Typhimurium are effective nanocarriers for delivering the hydrophobic compound violacein to cancer cells. These OMV enhance the cytoplasmic delivery of violacein and stimulate the accumulation of M1-type macrophages and the production of inflammatory mediators within the tumor microenvironment. The use of OMV to deliver violacein potentiates its antitumor effects and potentially reprograms the tumor microenvironment, all while maintaining a favorable safety profile. Thus, OMV from *S. enterica* Typhimurium loaded with anticancer, highly hydrophobic compounds represent a promising and cost-effective strategy for cancer therapy.

## Supplementary Material



## Notes

Estimated amount of violacein in μM according to the concentration of OMV/mL by HPLC area of standard violacein (Table S1); Transformation of bacterial strains with plasmid pBAT_*vioABCDE* containing the violacein operon (Figure S1); NTA of OMV isolated from culture supernatants of *S. enterica* Typhimurium (Figure S2); Relative intensity of violacein-loaded OMV by fluorescence microscopy (Figure S3); Weight of livers and spleens of mice inoculated with different doses of OMV (Figure S4); The safety evaluation of OMV of *S. enterica* Typhimurium by the subcutaneous route (Figure S5); Histological analysis of tumor sections of mice treated with OMV or violacein by the subcutaneous route (Figure S6); Histological analysis of organs of mice treated with OMV by the subcutaneous route (Figure S7) (PDF)
