# Supplementary material for: Violacein-Loaded Outer Membrane Vesicles from Salmonella enterica Exhibit Potent Anti-Melanoma Activity in Vitro and in Vivo
Source: ACS Biomater Sci Eng. 2025 Sep 11;11(10):6166–84. doi: 10.1021/acsbiomaterials.5c00933 (PMC12522090; doi:10.1021/acsbiomaterials.5c00933)
Supplement: Supplementary file 1 [file ab5c00933_si_001.pdf]

Supporting Information for Publication

Violacein-Loaded Outer Membrane Vesicles from *Salmonella enterica* Exhibit Potent Anti-Melanoma Activity *in Vitro and in Vivo*

Genesy Pérez Jorge[a,b*], *Marco Gontijo*[a,c], *Marina Flóro E Silva*[a,d], *Raquel Bester Liszbinski*[e,f], *Renata Spagolla Napoleão Tavares*[e], *Cyro von Zuben de Valega Negrão*[e,f], *Carlismari Oliveira Grundmann*[g], *Isabella Carolina Rodrigues dos Santos Goes*[a], *Lilian de Oliveira Coser*[h], *Elizabeth Bilsland*[i], *Francisca Janaína Soares Rocha*[j], *Monica Tallarico Pupo*[g], *Selma Giorgio*[d], *Sandra Martha Gomes Dias*[e], *Fausto Almeida*[k], *Marcelo Brocchi*[a]*

a Departamento de Genética, Evolução, Microbiologia e Immunologia, Instituto de Biologia, Universidade Estadual de Campinas – UNICAMP, 13083-862, Campinas, São Paulo, Brazil

b Research Group Statistics and Mathematical Modelling Applied to Educational Quality (GEMMA), University of Sucre, 700001, Sincelejo, Sucre, Colombia

c Department of Molecular Genetics and Microbiology, Duke University, NC 27710, Durham, USA

d Departamento de Biologia Animal, Instituto de Biologia, Universidade Estadual de Campinas - UNICAMP, 13083-862, Campinas, São Paulo, Brazil.

e Brazilian Biosciences National Laboratory (LNBio), Brazilian Center for Research in Energy and Materials (CNPEM), 13083-970, Campinas, São Paulo, Brazil.

f Graduate Program in Genetics and Molecular Biology, Institute of Biology, University of Campinas - UNICAMP, 13083-862, Campinas, São Paulo, Brazil

g School of Pharmaceutical Sciences of Ribeirão Preto, University of São Paulo, 14040-903, Ribeirão Preto, São Paulo, Brazil.

h Departamento de Patologia Clínica, Faculdade de Ciências Médicas, Universidade Estadual de Campinas - UNICAMP, 13083-887, Campinas, São Paulo, Brazil.

i Synthetic Biology Laboratory, Department of Structural and Functional Biology, Institute of Biology, Universidade Estadual de Campinas - UNICAMP, 13083-887, Campinas, São Paulo, Brazil.

j Tropical Medicine Department, Medical Science Center, Federal University of Pernambuco, 50670-901, Recife, Pernambuco, Brazil.

k Department of Biochemistry and Immunology, Ribeirão Preto Medical School, University of São Paulo, 14049-900, São Paulo, Brazil.

*GPJ: g211546@dac.unicamp.br genesyperezj@gmail.com

MTPG: marco.pardinigontijo@duke.edu

MFS: m261521@dac.unicamp.br

RBL: raquelb.biomed@gmail.com

RSNT: renata.tavares@lnbio.cnpem.br

CVZVN: cyro.negrao@lnbio.cnpem.br

COG: carlismari_grundmann@hotmail.com

ICRSG: i265705@dac.unicamp.br

LOC: l190135@dac.unicamp.br

EB: bilsland@unicamp.br

FJSR: janaina.srocha@ufpe.br

MTP: mtpupo@fcfrp.usp.br

SG: sgiorgio@unicamp.br

SMD: sandra.dias@lnbio.cnpem.br

FA: fbralmeida@usp.br

*MB: mbrocchi@unicamp.br

Supplementary Table

Table S1. Estimated amount of violacein in µM according to the concentration of OMV/ml by HPLC area of standard violacein.

Relative amount of violacein based on HPLC curve		
OMV/ml	*tolRAvio*-OMV	ST*vio*-OMV
7.95 x10^{7}	0.013766	0.003436
1.59x10^{8}	0.027521	0.006869
3.18x10^{8}	0.055041	0.013738
6.36x10^{08}	0.110082	0.027476
1.27x10^{09}	0.219818	0.054865
2.55x10^{09}	0.441368	0.110162
5.09x10^{09}	0.881004	0.219892
1.02x10^{10}	1.765471	0.440648
2.04x10^{10}	3.530941	0.881297
4.08x10^{10}	7.061882	1.762593
8.16x10^{10}	14.12376	3.525186

Supplementary figures

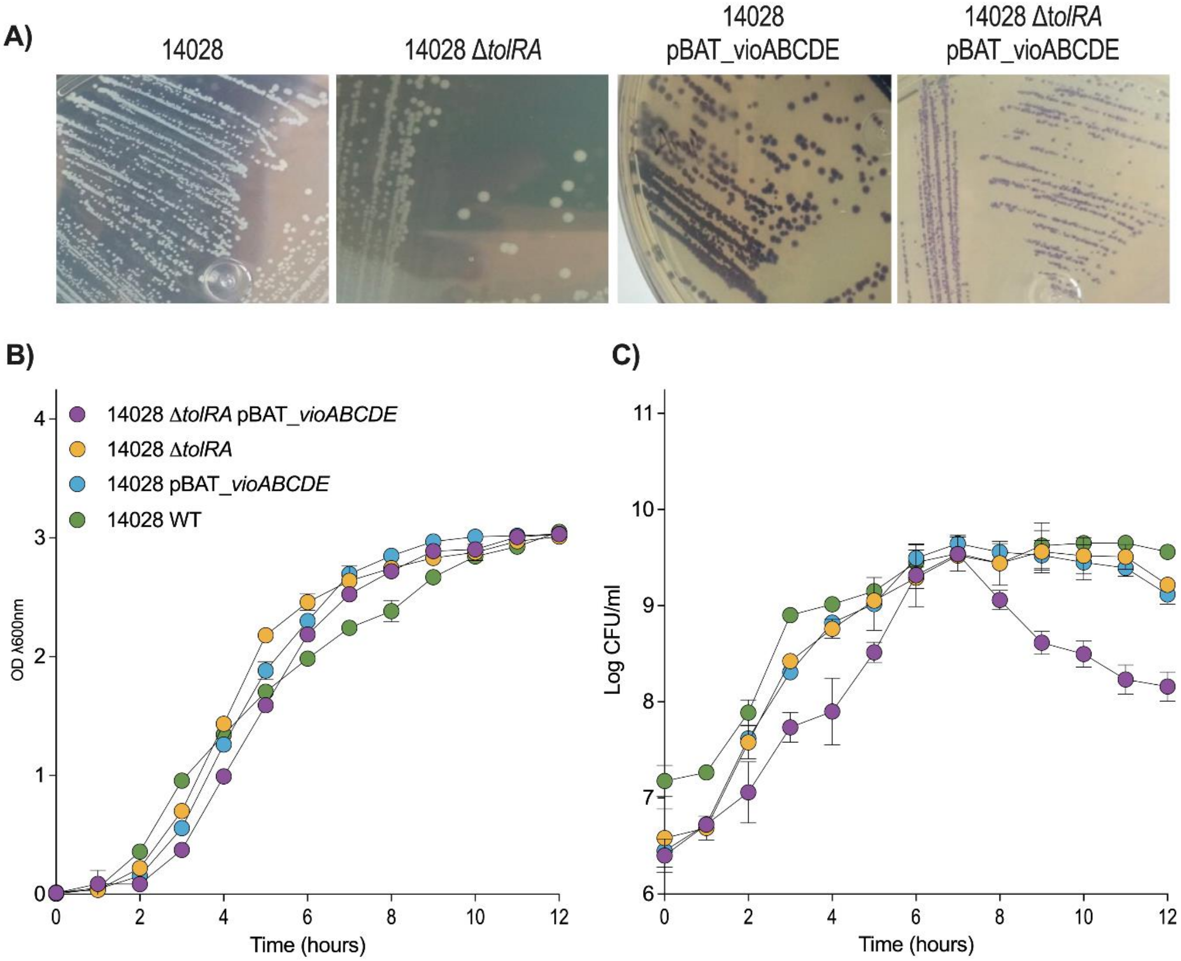

Figure S1. Transformation of bacterial strains with plasmid pBAT_*vioABCDE* containing the violacein operon. (A) Growth of *S. enterica* Typhimurium 14028 Δ*tolRA* and *S. enterica* Typhimurium 14028 Δ*tolRA* pBAT_*vioABCDE* mutants after 24 hours on LB agar plates and comparison with the parental strain. Colonies of the strain *S.* Typhimurium 14028 pBAT_*vioABCDE* are dark purple, while *S.* Typhimurium 14028 Δ*tolRA* pBAT_*vioABCDE* colonies are light purple. Bacterial growth was monitored for 12 hours from OD measurement (λ 600 nm) (B) and CFU count (C). This experiment was repeated three times independently with similar results.

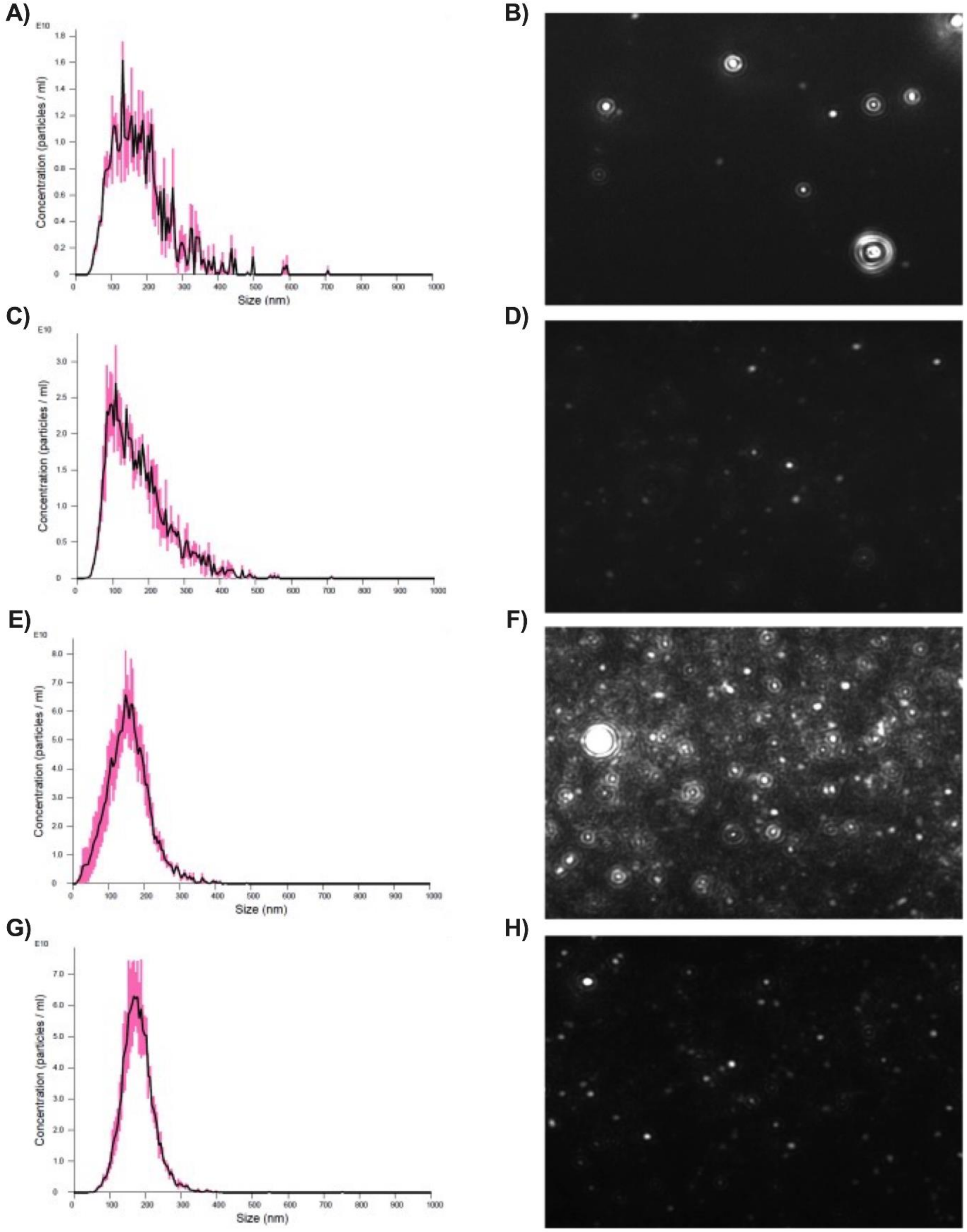
A)
B)
C)
D)
E)
F)
G)
H)
E10
Concentration (particles / ml)
Size (nm)

Figure S2. NTA of OMV isolated from culture supernatants of *S. enterica* Typhimurium. (A) and (B) histogram showing the particle size distribution and a video screenshot of ST-OMV. (C) and (D) histogram showing the particle size distribution and video screenshot of ST*vio*-OMV. (E) and (F) histogram showing the particle size distribution and video screenshot of *tolRA*-OMV, respectively. (G) and (H) histogram showing the particle size distribution and video screenshot of *tolRAvio*-OMV, respectively.

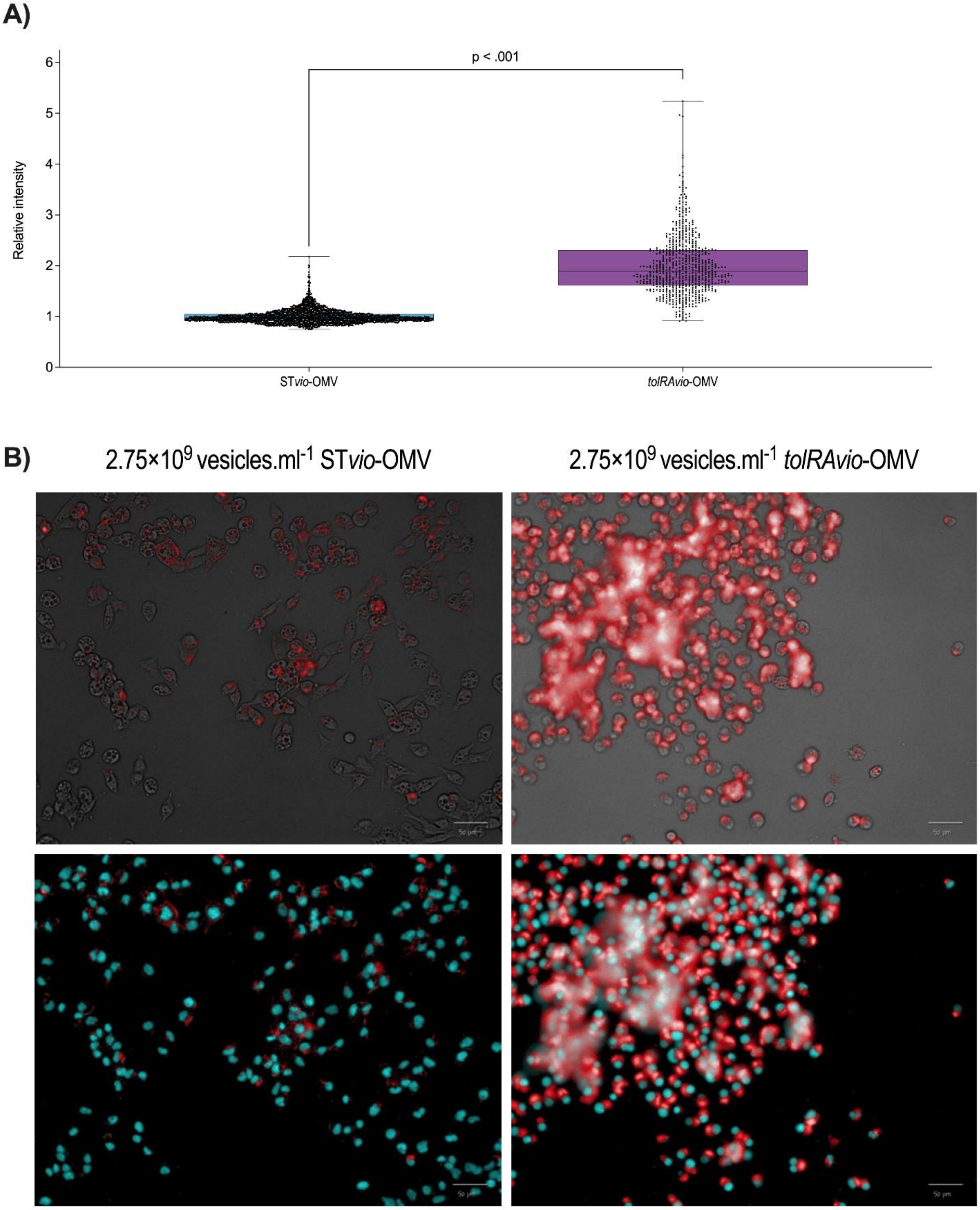
A)
p < .001
Relative intensity
0
1
2
3
4
5
6
STvio-OMV
tolRAvio-OMV
B)
2.75×10^9 vesicles.ml^-1 STvio-OMV
2.75×10^9 vesicles.ml^-1 tolRAvio-OMV

Figure S3. Relative intensity of violacein-loaded OMV by fluorescence microscopy. (A) B16-F10 cells after 4 hours of exposure to 2.75×10^9 vesicles /ml of ST*vio*-OMV or *tolRAvio*-OMV. Data are presented as mean valued ±SD. Statistical significance was determined by the Two-tailed Mann-Whitney test. (B) Representative images of B16-F10 cells exposure to *tolRAvio*-OMV or ST*vio*-OMV, top: bright field, bottom: dark field.

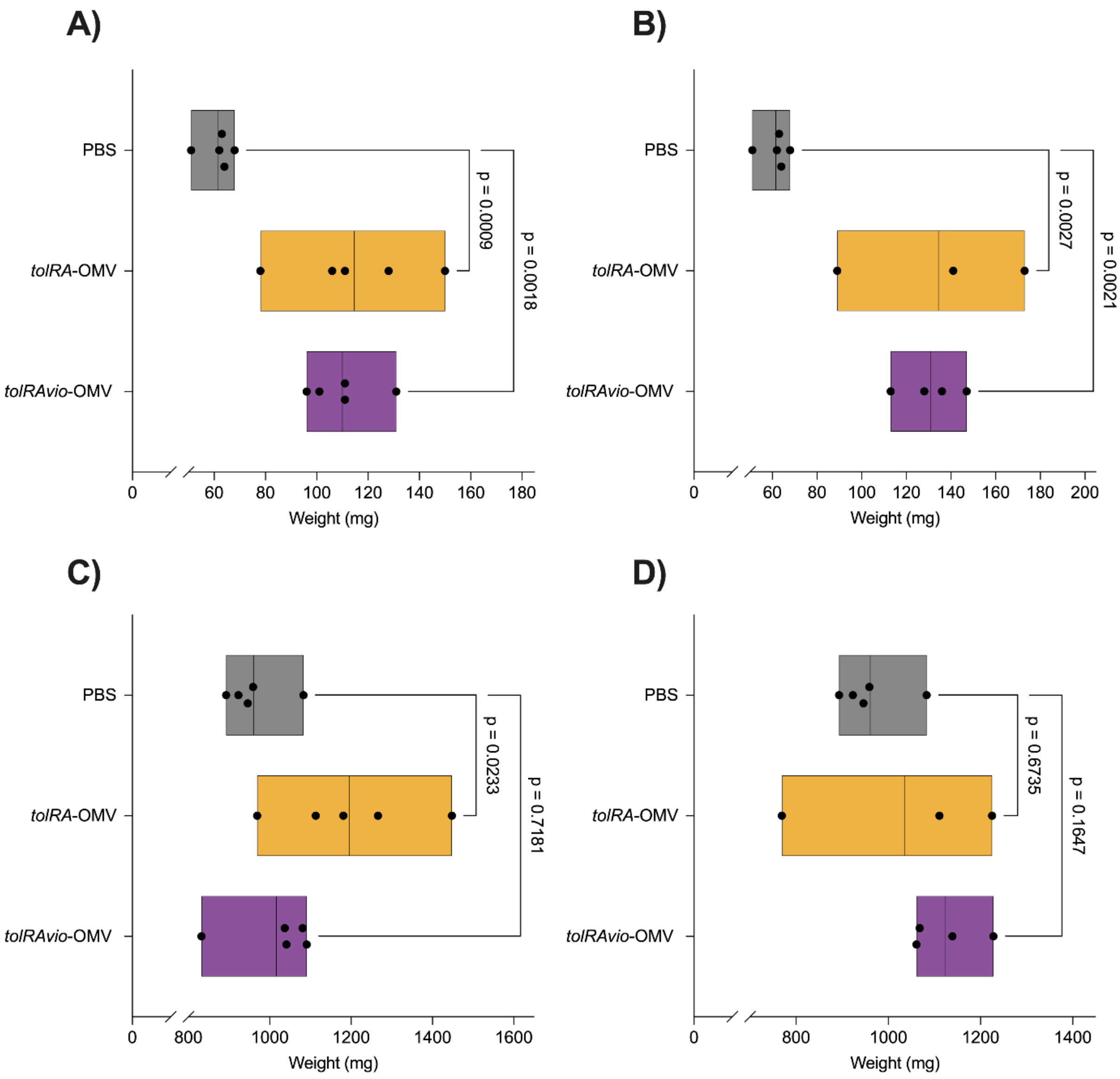

Figure S4. Weight of livers and spleens of mice inoculated with different doses of OMV. (A) Weight of spleens from mice inoculated with 1×10^{10} OMV. (B) Weight of spleens from mice inoculated with 2×10^{10} OMV. (C) Weight of livers from mice inoculated with 1×10^{10} OMV. (D) Weight of livers from mice inoculated with 2×10^{10} OMV. Statistical significance was calculated by one-way ANOVA. This experiment was repeated two times independently with similar results.

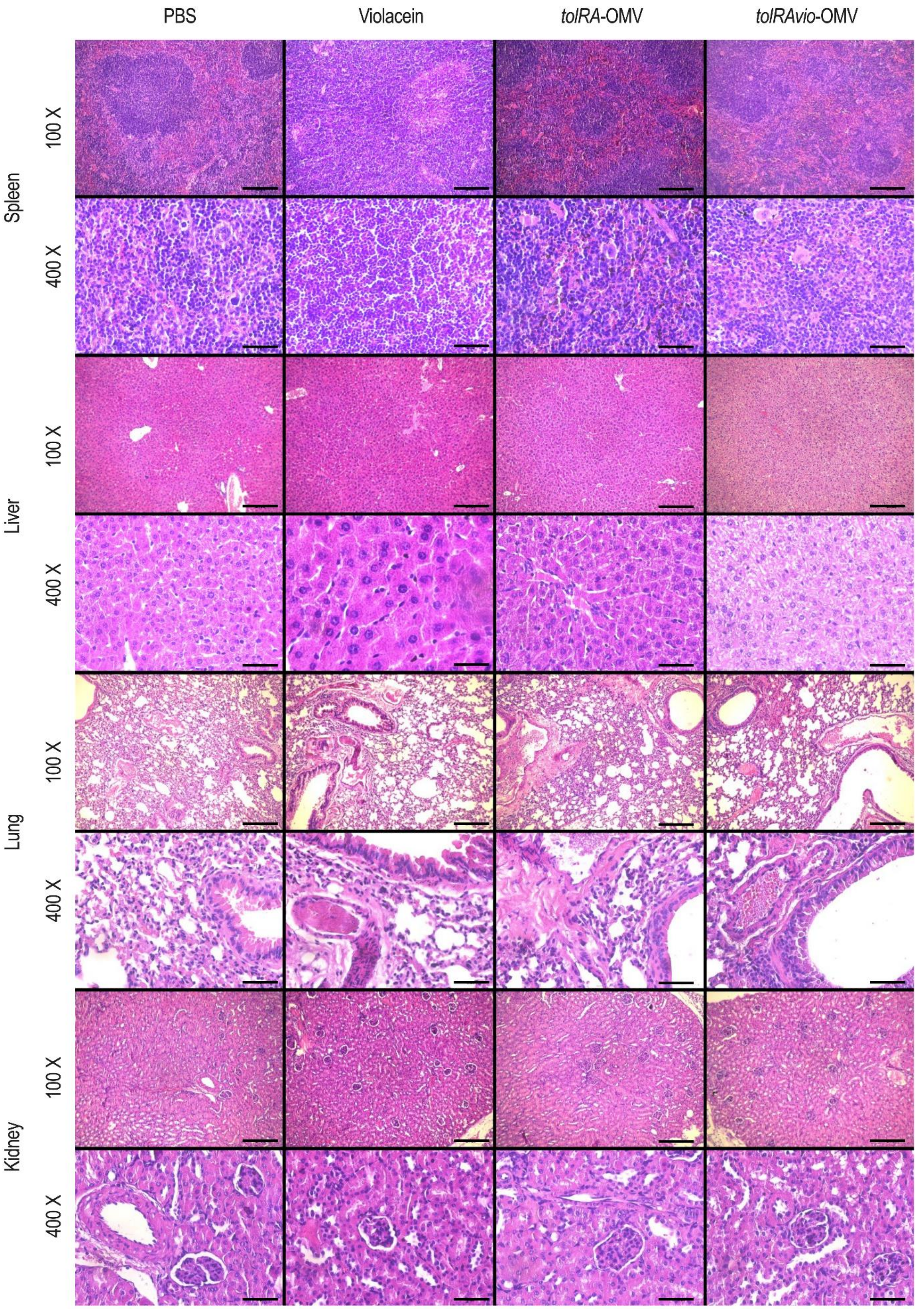
PBS
Violacein
tolRA-OMV
tolRAvio-OMV
Spleen
Liver
Lung
Kidney
100 X
400 X

83

Figure S5. The safety evaluation of OMV of *S. enterica* Typhimurium by the subcutaneous route. Healthy C57BL/6Junib mice were inoculated with 5×10^9 ST-OMV, ST*vio*-OMV, *tolRA*-OMV, or *tolRAvio*-OMV twice a week for two weeks. The liver, spleen, lung, and kidney were collected and analyzed by H&E staining. In the spleens of mice inoculated with *tolRA*-OMV or *tolRAvio*-OMV, hypertrophy and increased megakaryocytes were observed. In the livers of mice treated with *tolRA*-OMV or *tolRAvio*-OMV, inflammatory cell infiltration was observed without hepatocyte degeneration. Scale bar: 200 μm for ×100 and 50 μm for ×400.

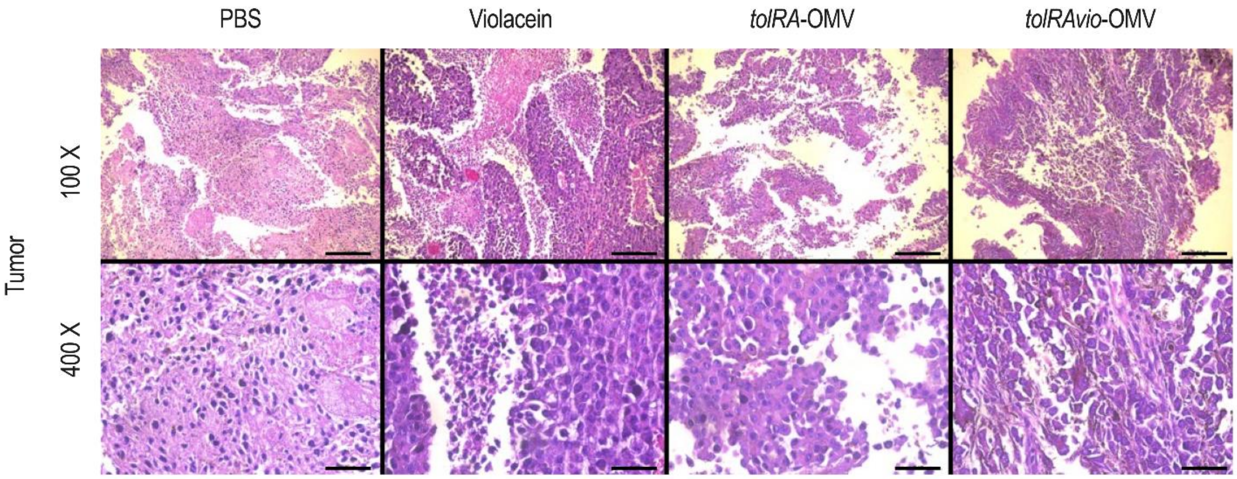

Figure S6. Histological analysis of tumor sections of mice treated with OMV or violacein by the subcutaneous route. Tumor-bearing C57BL/6Junib mice were treated with 5×10^9 of *tolRA*-OMV or *tolRAvio*-OMV or violacein (306 ng) twice a week for two weeks. Tumors were collected and analyzed by H&E staining. Tumor sections from control group mice (PBS) reveal destruction of the epidermis, abundance of necrotic regions, and abundant neoplastic cells. Some apoptotic cells were observed in the group treated with violacein, which was more abundant in the groups treated with *tolRA*-OMV or *tolRAvio*-OMV. Tumor necrosis and inflammatory infiltration were also observed in these two groups. Scale bar: 200 µm for ×100 and 50 µm for ×400.

103

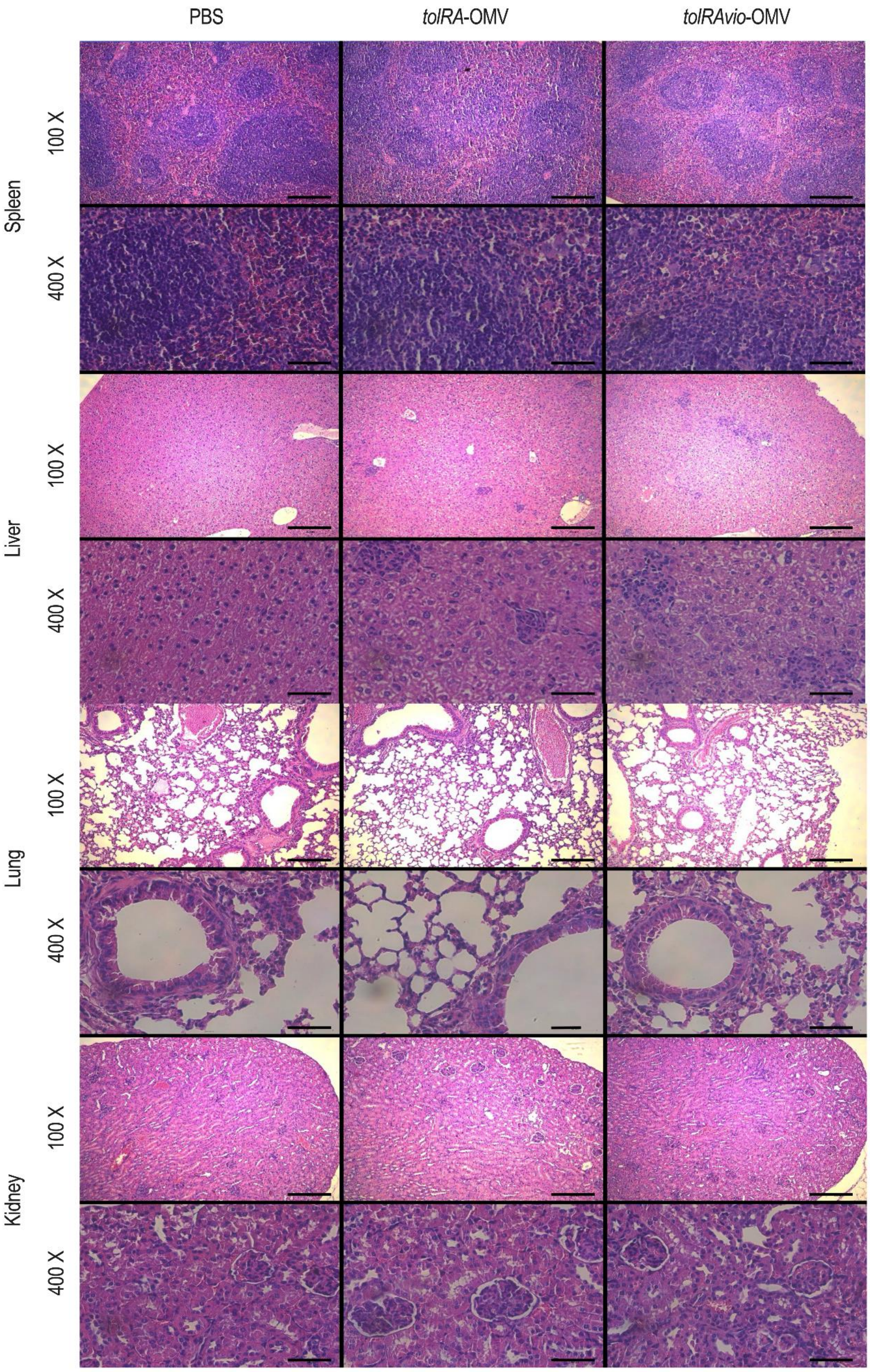
PBS
tolRA-OMV
tolRAvio-OMV
Spleen
100 X
400 X
Liver
100 X
400 X
Lung
100 X
400 X
Kidney
100 X
400 X

Figure S7. Histological analysis of organs of mice treated with OMV by the subcutaneous route. Tumor-bearing C57BL/6Junib mice were treated with 5×10^9 of *tolRA*-OMV or *tolRAvio*-OMV twice a week for two weeks. The liver, spleen, lung, and kidney were collected and analyzed by H&E staining. No significant organ damage was observed in mice treated with violacein, *tolRA*-OMV, or *tolRAvio*-OMV. Scale bar: 200 µm for ×100 and 50 µm for ×400.
